# Prenatal Intravenous Iron and Child Growth: A Secondary Analysis of a Randomized Clinical Trial

**DOI:** 10.1001/jamanetworkopen.2025.38392

**Published:** 2025-10-28

**Authors:** Glory Mzembe, William Nkhono, Ernest Moya, Rebecca Harding, Justina Kaunda, Alex Thawani, Mphatso Mwambinga, Owen Mtambo, Zinenani Truwah, Maclean Vokhiwa, Gomezgani Mhango, Ayşe Y. Demir, Hans Verhoef, Ricardo Ataíde, Sabine Braat, Sant-Rayn Pasricha, Kamija S. Phiri, Martin N. Mwangi

**Affiliations:** 1 Training and Research Unit of Excellence, Blantyre, Malawi; 2 School of Global and Public Health, Department of Public Health, Kamuzu University of Health Sciences, Blantyre, Malawi; 3 Infection and Global Health Division, The Walter and Eliza Hall Institute of Medical Research, Melbourne, Australia; 4 Department of Infectious Diseases, University of Melbourne, at the Peter Doherty Institute for Infection and Immunity, Melbourne, Australia; 5 Centre for Epidemiology and Biostatistics, Melbourne School of Population and Global Health, The University of Melbourne, Melbourne, Australia; 6 Laboratory for Clinical Chemistry and Haematology, Meander Medical Centre, Amersfoort, the Netherlands; 7 Division of Human Nutrition and Health, Wageningen University, Wageningen, the Netherlands; 8 Diagnostic Haematology, The Royal Melbourne Hospital, Parkville, Victoria, Australia; 9 Clinical Haematology, The Peter MacCallum Cancer Centre and The Royal Melbourne Hospital, Parkville, Victoria, Australia; 10 Department of Medical Biology, University of Melbourne, Parkville, Victoria, Australia; 11 The Micronutrient Forum, Healthy Mothers Healthy Babies Consortium, Washington, DC

## Abstract

**Importance:**

Maternal anemia during pregnancy is associated with poor growth during early childhood, but it remains unclear whether treatment using intravenous iron could enhance child growth.

**Objective:**

To evaluate whether intravenous ferric carboxymaltose (FCM) treatment for moderate to severe anemia during the second trimester of pregnancy enhances postnatal child growth compared with standard-of-care (SOC) oral iron.

**Design, Setting, and Participants:**

This was a prespecified secondary analysis of a randomized clinical trial (Randomized Controlled Trial of the Effect of Intravenous Iron on Anaemia in Malawian Pregnant Women [REVAMP]). In REVAMP, pregnant women with capillary hemoglobin between 5 and 10 g/dL were recruited in the Blantyre and Zomba districts of southern Malawi between November 2018 and March 2022 and randomized to FCM or SOC. Their infants were followed up over 12 months post partum. Data analysis was conducted between August 2024 and August 2025.

**Intervention:**

Mothers were given intravenous FCM up to 1000 mg once on trial enrollment (intervention group) or SOC oral ferrous sulfate, 200 mg twice daily (approximately 120 mg elemental iron per day) (control group) from 13 to 26 weeks’ gestation until delivery. Children received no intervention.

**Main Outcomes and Measures:**

Length-for-age *z* score (LAZ) at 12 months was the primary growth outcome; secondary outcomes included weight-for-age *z* scores (WAZ), weight-for-length *z* scores (WLZ), stunting (LAZ <−2), underweight (WAZ <−2), and wasting (WLZ <−2). Length and weight were measured at birth and at 1, 3, 6, 9, and 12 months post partum. The repeated measures were analyzed using mixed-effects models.

**Results:**

A total of 738 infants were followed up for 12 months between March 2019 and October 2022. A total of 371 (193 [52.0%] male; mean [SD] gestational age at birth, 39.5 [1.9] weeks) were in the FCM group, and 367 (187 [51.2%] male; mean [SD] gestational age at birth, 39.4 [2.3] weeks) were in the SOC group. There was no evidence of a difference between FCM and SOC groups in mean LAZ (mean difference, −0.15 [95% CI, −0.37 to 0.08]), WAZ (mean difference, −0.02 [95% CI, −0.21 to 0.16]) or WLZ (mean difference, 0.02 [95% CI, −0.20 to 0.24]) at 12 months. Similarly, there was no difference in the proportion with stunting, underweight, or wasting at all time points. Stunting was more common in mothers younger than 20 years, less than 150 cm in height, and with no education or primary education only, although treatment effects showed no evidence of heterogeneity.

**Conclusions and Relevance:**

In this secondary analysis of a randomized clinical trial in southern Malawi, intravenous FCM treatment for moderate to severe anemia during the second trimester of pregnancy was not associated with substantial benefits on infant growth compared with SOC.

**Trial Registration:**

anzctr.org.au Identifier: ACTRN12618001268235

## Key Points

**Question:**

Compared with standard-of-care (SOC) oral iron, does intravenous ferric carboxymaltose (FCM) treatment for pregnant women with anemia enhance postnatal child growth?

**Findings:**

In this secondary analysis of a randomized clinical trial with 738 infants in southern Malawi, there was no evidence of a difference in infants’ length for age, weight for age, or weight for length between the 2 groups.

**Meaning:**

Compared with SOC, FCM treatment was not associated with substantial benefits on infant growth in southern Malawi.

## Teaser

This secondary analysis of a randomized clinical trial examines whether intravenous iron treatment vs standard-of-care oral iron improves infant growth among children whose mothers had moderate to severe anemia during the second trimester of pregnancy.

## Introduction

Child growth failure, including stunting (too short for age), underweight (too thin for age), and wasting (too thin for height), has declined over the past 2 decades but remains high in low- and middle-income countries (LMICs).^1^ Malawi is among LMICs with the largest number of growth failures, with nearly 4 in 10 children (38%) younger than 5 years stunted and 1 in 10 (10%) underweight, although wasting is less common, affecting less than 5%.^2^ While underweight and wasting are acute signs of impaired growth, stunting is a chronic indicator and is largely irreversible.^3,4^ Impaired growth in early life, particularly in the first 1000 days, is associated with increased morbidity and mortality in childhood, and particularly, stunting is associated with long-term detrimental effects, including poor cognitive development and reduced educational performance.^5,6,7,8,9^ Reducing growth failure is thus a major global health priority.^10^

Evidence indicates that growth failure starts during the fetal period, continuing through the first 2 years of age.^11^ Observational studies have linked maternal anemia during pregnancy to impaired child growth.^12,13,14^ Maternal anemia is associated with intrauterine growth restriction, preterm delivery, low birth weight, and reduced placental iron transfer to the fetus, thus, subsequent inferior health and impaired growth.^15^ Anemia is widespread in sub-Saharan Africa (SSA), affecting as much as 50% of pregnancies.^16^ Iron deficiency is considered the major cause; thus, the World Health Organization (WHO) recommends daily oral iron supplementation throughout pregnancy in this setting. While some studies have shown a reduction in the incidence of stunting in infants associated with maternal iron supplementation,^17,18,19^ the finding has not been consistently reproduced.^20,21^ The discrepancy has been mainly attributed to low adherence to supplementation and inadequate absorption due to infection-related inflammation.^17,22,23^ Intravenous (IV) iron addresses adherence challenges and rapidly improves maternal iron stores, yet its effect on infant growth is largely unexamined.^24,25^

We conducted a randomized clinical trial in southern Malawi (Randomized Controlled Trial of the Effect of Intravenous Iron on Anaemia in Malawian Pregnant Women [REVAMP]),^26,27^ which provided an opportunity to investigate the effect of prenatal IV iron on infant growth. Malawi is a malaria-endemic and infectious disease–prone setting where up to 45% of pregnant women have anemia.^2^ Iron deficiency ranges from 44% to 61%.^28^ Adherence to the full course of standard-of-care (SOC) oral iron is very low, estimated at 33%.^22^ We hypothesized that using IV iron would enhance infant postpartum growth. The REVAMP trial showed a reduction in the prevalence of iron deficiency in women assigned to IV ferric carboxymaltose (FCM), compared with SOC (FCM, 60 of 336 participants [18%]; SOC, 142 of 341 participants [42%]; prevalence ratio, 0.43 [95% CI, 0.33-0.55]), but there was no significant difference in anemia prevalence at 36 weeks’ gestation, although prevalence rates were lower in the FCM group.^27^ This study investigates whether IV FCM treatment during the second trimester of pregnancy enhances infant growth outcomes up to 12 months, filling a critical evidence gap on a major obstacle to human development.

## Methods

### Study Design, Setting, and Participants

This prespecified secondary analysis was a prospective longitudinal study that tracked growth from birth to 12 months of age in a cohort of infants born to mothers participating in an open-label, individual-randomized clinical trial (REVAMP), conducted in Blantyre and Zomba districts, southern Malawi, between November 2018 and March 2022.^26,27^ The primary end point of the REVAMP trial was anemia prevalence at 36 weeks’ gestation, with secondary end points in mother and infant up to 1 month post partum. The trial protocol and statistical analysis plan appear in Supplement 1.

This follow-up study was conducted between March 2019 and October 2022. All live infants born to women enrolled in the REVAMP trial were eligible to take part but could opt out during a written informed consent process, conducted either at trial baseline for the full duration of follow-up or consented until 1 month post partum, then reconsented for the extended follow-up after 1 month post partum. As not all participants gave consent for the extended follow-up, participants included in this study are a subset of the REVAMP trial. The REVAMP trial, including this extended follow-up, were approved by ethics committees at the Kamuzu University of Health Sciences, Malawi; and WEHI, Australia, monitored by an independent data and safety monitoring board, and prospectively registered (ACTRN12618001268235). This report follows the Consolidated Standards of Reporting Trials (CONSORT) reporting guideline.

### Intervention and Exposure

Full details of the REVAMP protocol have been published previously.^26^ Briefly, women were eligible if they had capillary hemoglobin concentration of less than 10.0 g/dL but greater than 5.0 g/dL by HemoCue 301+ (to convert to grams per liter, multiply by 10), were *Plasmodium falciparum* negative by rapid diagnostic test (or microscopy if previously rapid test positive and treated), had an ultrasonography-confirmed singleton pregnancy at 13 to 26 weeks’ gestation, and were not clinically considered to urgently require transfusion or hospitalization. Eligible women were randomized (by sealed envelope) in a 1:1 ratio with randomly permuted blocks of size 4 or 6, stratified by site to either FCM (Vifor Pharma) 20 mg/kg, up to 1000 mg, diluted in 250 mL normal saline, intravenously over 15 minutes on trial enrollment or to SOC, 200 mg of oral ferrous sulfate (equivalent of 65 mg elemental iron) twice daily until delivery. All participants received intermittent preventive treatment with sulfadoxine-pyrimethamine for malaria unless HIV positive and receiving cotrimoxazole.

### Study Outcomes

The primary growth outcome of this longitudinal follow-up study was length-for-age *z* score (LAZ) at 12 months. Secondary outcomes included weight-for-age *z* scores (WAZ), weight-for-length *z* scores (WLZ), stunting (LAZ <−2), underweight (WAZ <−2), and wasting (WLZ <−2).

### Data Collection

Length and weight measurements were recorded at the study clinic at birth and at 1, 3, 6, 9, and 12 months of age. Trained research nurses measured child length using a Seca kiddimetre (Seca GmbH & Co; precision, 1 mm) and weight with a Seca scale (Seca GmbH & Co; precision, 10 g). Length was measured with the infant in a supine position, with the knee extended and heels and head touching the bases of the kiddimetre. Each measurement was taken twice, and a third one was taken if the difference between the first 2 exceeded 5%. The mean of the 2 closest values was used for analysis. Birth weight and birth length were collected within 24 hours of birth. Data were collected and managed using Open Data Kit.

### Statistical Analysis

The sample size was determined by the REVAMP trial, which aimed to detect a 10% reduction in anemia prevalence at 36 weeks’ gestation (60% SOC vs 50% FCM) in 862 women (90% power, 2-sided α = .05) with an assumed 10% loss to follow up.^29^ The analysis sample included all liveborn infants whose mothers consented to participate in the extended follow-up with at least 1 nonmissing outcome value. Infant outcomes were analyzed according to the randomly allocated group of the mother, and results are presented for all time points from birth to 12 months of age. Twins were included in the analysis, but due to the low proportion, we did not account for any clustering in the statistical models.

We derived *z* scores using the Stata Macro of the WHO Anthro Software and the WHO Anthro Survey Analyzer (version 3.2.2; January 2011), which adjusts for age (in days) and sex. We analyzed LAZ, WAZ, and WLZ using a linear mixed model by Liang and Zeger,^30^ with a random intercept for participant and an unstructured variance-covariance among the repeated measurements. Stunting, underweight, and wasting were defined as *z* scores below −2 for LAZ, WAZ, and WLZ, respectively, Stunting, underweight, and wasting were analyzed using mixed-effects Poisson regression models with a log link and robust SEs to directly estimate the risk ratio. The fixed effects consisted of treatment, study visit (categorical), treatment × study visit interaction, and site (the randomization stratification factor).^31^ These longitudinal models were fitted to all study visits from birth to 12 months of age. Results are presented as absolute mean differences or risk ratios of FCM to SOC with 2-sided 95% CIs, together with their associated *P* values, at each study visit. *P* < .05 was considered statistically significant. We performed similar analyses using *z* scores derived using the Intergrowth-21st gigs Stata package,^32^ which adjusts for gestation age in preterm infants, and excluding implausible values using the cutoffs recommended by the WHO, which include LAZ less than –6 or greater than 6, WAZ less than –6 or greater than 5, and WLZ less than –5 or greater than 5,^33^ to explore the sensitivity of the results.

Additionally, for each outcome measure, we conducted subgroup analyses using the previously described models and incorporating a fixed effect of subgroup and subgroup × treatment × visit interaction and subgroup × treatment and subgroup × visit interaction for a prespecified list of factors known to affect treatment outcomes and infant growth from the literature. These included maternal baseline iron status (iron deficient or not iron deficient), inflammation (yes or no), placental malaria parasitemia (positive or negative), HIV status (positive or negative), age (<20 years or ≥20 years), height (<150 cm or ≥150 cm), education (none and primary or secondary and tertiary), and infant sex (male or female). The purpose of the interaction testing was exploratory rather than confirmatory. Results are presented as absolute mean differences or risk ratios with their 2-sided 95% CI and *P* value for each subgroup, in addition to interaction *P* value.

The statistical models use a likelihood-based approach to handle missing data that assumes missingness is at random among those participants with available data; thus, missing values were not imputed for any analyses. No adjustments were made for multiple comparisons. All statistical analyses were performed in Stata version 16.0 (StataCorp).

## Results

### Characteristics of the Study Population

**Figure 1.**
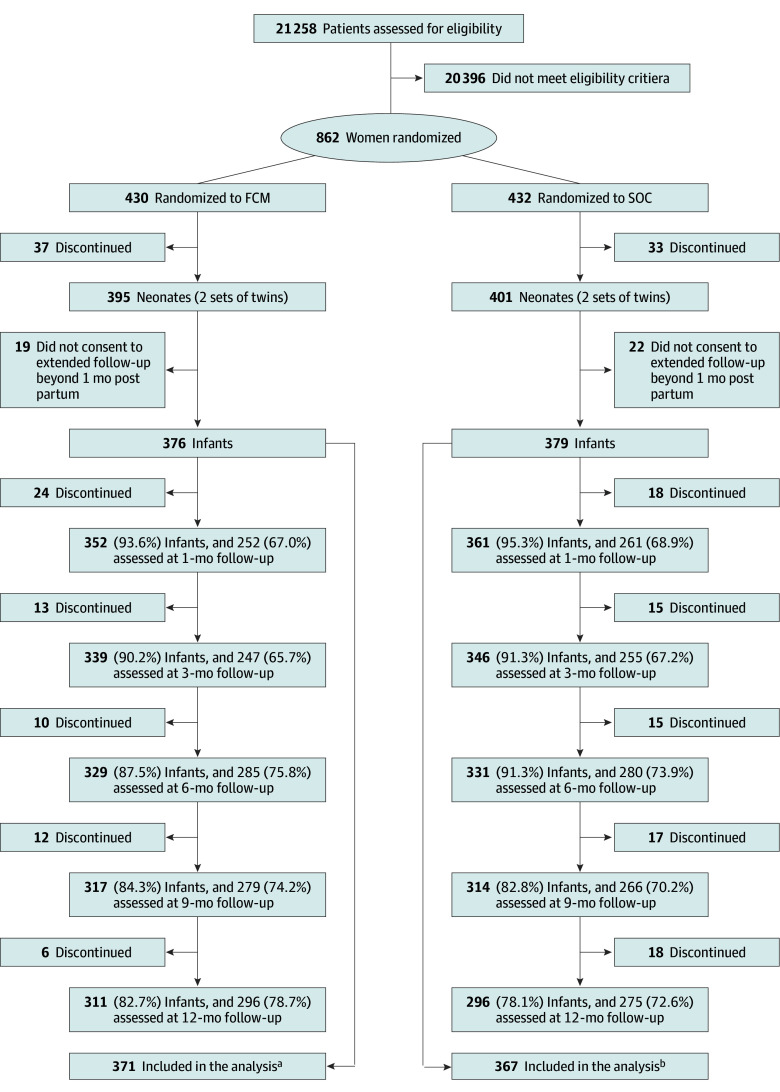
Study Flow Diagram Missing data were mainly due to the COVID-19 pandemic and lockdowns, which affected participants attendance of the study visits. Additionally, for 1-month and 3-month visits, delays in ethics approval to extend the trial follow-up and add infant growth as one of the secondary objectives of the trial meant some participants were systematically missed. These participants were already older than 3 months when they were reconsented for the extended follow-up. FCM indicates ferric carboxymaltose; SOC, standard of care. ^a^A total of 5 infants were excluded because they did not have growth data at any time point. ^b^A total of 12 infants were excluded because they did not have growth data at any time point.

The extended follow-up was conducted between March 2019 and October 2022. A total of 796 liveborn infants (395 in FCM group; 401 in SOC group) were recorded (4 sets of twins; 2 pairs of twins in each treatment group). Mothers of 41 infants (5.2%; 19 in FCM and 22 in SOC) did not consent to the extended follow-up beyond 1 month post partum. Of the remaining 755, 17 (2.3%) were excluded as they did not have data available; thus, 738 infants, 371 (193 [52.0%] male; mean [SD] gestational age at birth, 39.5 [1.9] weeks) in the FCM group and 367 (187 [51.2%] male; mean [SD] gestational age at birth, 39.4 [2.3] weeks) in the SOC group, were included in the analysis (Figure 1).

The distribution of maternal characteristics at trial enrollment was similar between the 2 treatment groups (Table 1). The mean (SD) maternal age for the study population was 22.4 (6.3) years, with 330 of 734 mothers (45.0%) younger than 20 years. Mean (SD) height was 155.3 (6.3) cm, and 123 (16.8%) had a height below 150 cm. Slightly more than half (395 [53.8%]) were pregnant for the first time, and 30.5% (224 mothers) were enrolled after malaria treatment. The highest level of education for most was primary school (454 of 708 [64.1%]), with less than 2% (14 mothers) having attained tertiary education. The prevalence of maternal baseline iron deficiency (303 of 717 [42.3%]) and inflammation (370 of 717 [51.6%]) was similar between groups. Mean (SD) gestational age at birth was 39.5 (2.1) weeks, while mean (SD) birth weight was 2893 (509) g, and both were comparable between the 2 groups. Breastfeeding was high (>95%) and comparable between groups at all the time points.

**Table 1.** Characteristics of Participating Mothers and Infants^a^

Characteristic	Participants, No./total No. (%)	
	FCM (n = 371)	SOC (n = 367)
**Maternal characteristics**		
Age, y		
Mean (SD)	22.2 (6.3)	22.5 (6.4)
<20	165/369 (44.7)	165/365 (45.2)
Height, cm		
Mean (SD)	155.3 (5.7)	155.2 (6.8)
<150 cm	57/369 (15.5)	66/365 (18.1)
Weight at randomization, mean (SD), kg	55.8 (8.1)	55.1 (8.1)
BMI at randomization		
Mean (SD)	23.1 (3.0)	22.9 (2.9)
Underweight (BMI <18.5)	8/369 (2.2)	12/365 (3.3)
Normal weight (BMI 18.5 to <25.0)	284/369 (77.0)	279/365 (76.4)
Overweight (BMI 25.0 to <30.0)	66/369 (17.9)	68/365 (18.6)
Obesity (BMI ≥30.0)	11/369 (3.0)	6/365 (1.6)
Gestation age at randomization, mean (SD), wk	21.7 (3.2)	21.4 (3.3)
Primigravid^b^	194/369 (52.6)	201/365 (55.1)
Marital status^b^		
Single	55/368 (15.0)	62/363 (17.1)
Married	309/368 (84.0)	290/363 (79.9)
Widowed	0/368	3/363 (0.8)
Divorced or separated	4/368 (1.1)	8/363 (2.2)
Education^b^		
None	1/357 (0.3)	1/351 (0.3)
Primary	231/357 (64.7)	221/351 (63.0)
Secondary	117/357 (32.8)	123/351 (35.0)
Tertiary	8/357 (2.2)	6/351 (1.7)
Income source^b^		
None	20/368 (5.4)	28/363 (7.7)
Subsistence farming	75/368 (20.4)	65/363 (17.9)
Large-scale farming	1/368 (0.3)	1/363 (0.3)
Employed	62/368 (16.9)	52/363 (14.3)
Casual work for wages	120/368 (32.6)	110/363 (30.3)
Business	86/368 (23.4)	103/363 (28.4)
Other	4/368 (1.1)	4/363 (1.1)
HIV status, positive	61/367 (16.6)	62/361 (17.2)
Malaria at screening, positive^c^	116/369 (31.4)	108/365 (29.6)
Hemoglobin at randomization, mean (SD), g/dL	8.8 (1.2)	8.9 (1.2)
Anemia at randomization^d^		
No anemia (hemoglobin ≥11 g/dL)^e^	15/365 (4.1)	17/365 (4.7)
Mild anemia (hemoglobin 10 g/dL to <11 g/dL)	37/365 (10.1)	45/365 (12.3)
Moderate anemia (hemoglobin 7 g/dL to <10 g/dL)	288/365 (78.9)	278/365 (76.2)
Severe anemia (hemoglobin <7g/dl)	25/365 (6.9)	25/365 (6.9)
Ferritin at randomization, median (IQR), µg/L	26.4 (9.9-77.8)	30.1 (11.0-70.9)
Iron deficiency at randomization^f^	157/358 (43.9)	146/359 (40.7)
Iron deficiency anemia at randomization^f^	147/354 (41.5)	141/359 (39.0)
Inflammation at randomization^g^	185/358 (51.7)	185/359 (51.5)
Anemia and inflammation at randomization^g^	177/354 (50.0)	175/359 (48.8)
Hemoglobin at delivery (venous), mean (SD), g/dL	11.8 (1.6)	11.7 (1.6)
Anemia at delivery^d^	89/339 (26.3)	103/330 (31.2)
Positive placental malaria parasitemia^h^	112/313 (35.8)	115/304 (37.8)
**Infant characteristics at delivery**		
Sex		
Male	193/371 (52.0)	187/365 (51.2)
Female	178/371 (48.0)	178/365 (48.8)
Gestational age at birth		
Mean (SD), wk	39.5 (1.9)	39.4 (2.3)
Preterm birth (<37 wk gestation)	31/371 (8.4)	35/367 (9.5)
Birth weight		
Mean (SD), g	2892 (501)	2894 (516)
Low birthweight (<2500 g)	65/370 (17.6)	58/362 (16.0)
Breastfeeding^i^		
At 1 mo	223/227 (98.2)	236/239 (98.7)
At 12 mo	210/218 (96.3)	193/197 (98.0)
Exclusive breastfeeding		
At 1 mo	218/227 (96.0)	231/239 (96.7)
At 3 mo	210/240 (87.5)	214/244 (87.7)
At 6 mo	23/257 (9.0)	22/255 (8.6)
At 9 mo	1/230 (0.4)	1/222 (0.5)

Abbreviations: BMI, body mass index (calculated as weight in kilograms divided by height in meters squared); FCM, ferric carboxymaltose; SOC, standard of care.

SI conversion factor: To convert hemoglobin to grams per liter, multiply by 10.

^a^ Includes all liveborn infants from the Randomized Controlled Trial of the Effect of Intravenous Iron on Anaemia in Malawian Pregnant Women (REVAMP) trial whose mothers consented to participate in the extended follow-up to 12 months of age and with at least 1 nonmissing outcome value. Of the 755 mothers who consented, 17 (2.3%) did not have data available at any 1 point in time.

^b^ Self-reported.

^c^ If women met the anemia criteria but had a positive malaria rapid diagnostic test, they were treated for malaria as per local protocols and deferred from enrollment. These women were able to present for rescreening no earlier than 7 days later and were enrolled if they met the eligibility criteria with malaria parasitemia assessed using microscopy.

^d^ Anemia indicates hemoglobin level of less than 11g/dL.

^e^ Venous hemoglobin analyzed by Sysmex (not capillary hemoglobin which was used on screening and recruitment) was used to ensure standardization. This explains why there are some participants with no or mild anemia recruited into the study though the criterion was moderate to severe anemia.

^f^ Maternal iron deficiency was defined as serum ferritin less than 15 µg/L or ferritin less than 30 µg/L if C-reactive protein was greater than 0.5 mg/dL (to convert to milligrams per liter, multiply by 10). Iron deficiency anemia indicates venous hemoglobin of less than 11 g/dL and serum ferritin less than 15 µg/L or ferritin less than 30 µg/L if C-reactive protein is greater than 0.5 mg/dL.

^g^ Inflammation indicates C-reactive protein greater than 0.5 mg/dL, and anemia and inflammation indicates venous hemoglobin of less than 11.0g/dL and C-reactive protein level greater than 0.5 mg/L.

^h^ Placental malaria parasitemia histology, denotes both past and active infection.

^i^ Denotes both exclusive and mixed breastfeeding.

### Infant Growth

Mean absolute length and weight were similar in both groups at all time points (eTable 1A in Supplement 2). Mean (SD) absolute length for the study population increased from 47.8 (3.1) cm at birth to 71.7 (3.5) cm at 12 months, while mean absolute weight increased from 2893 (508) g to 8526 (1179) g. Compared with the WHO reference population, participants in the study population were on average 1.8 cm shorter at birth and 3.2 cm shorter at 12 months and 396.3 g lighter at birth and 771.0 g lighter at 12 months. Sensitivity analyses excluding implausible values produced similar results (eTable 1B in Supplement 2).

**Figure 2.**
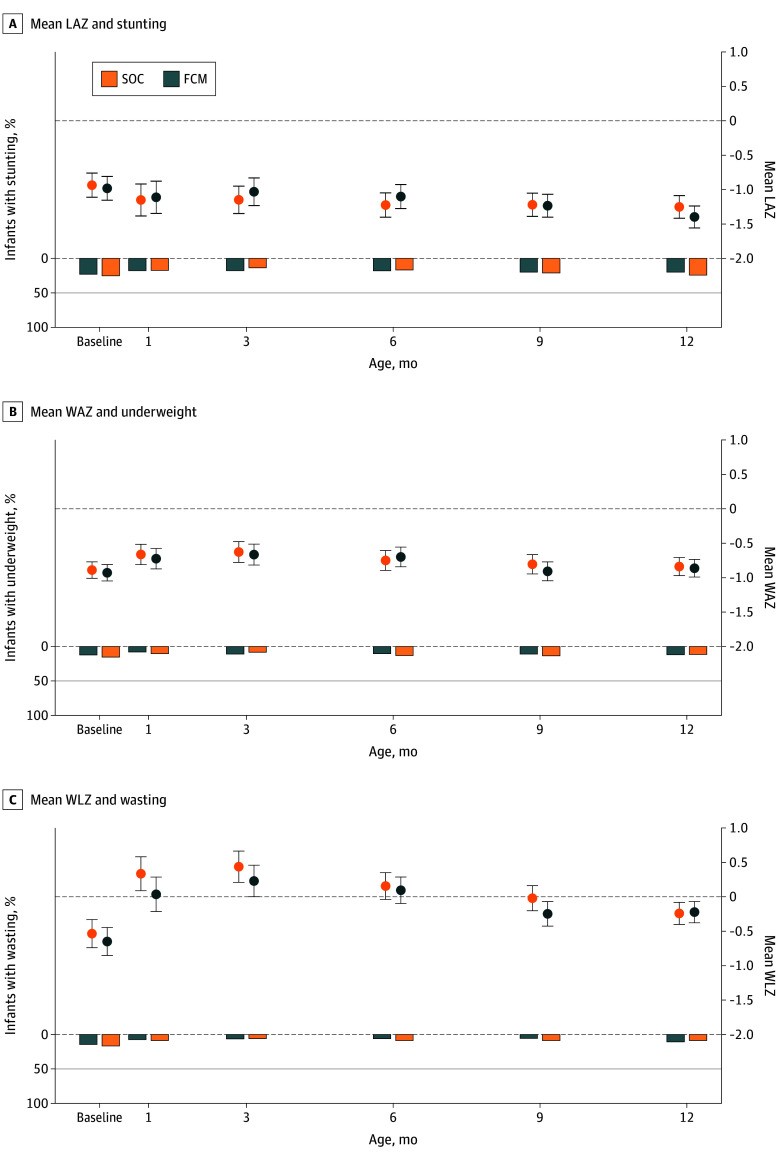
Mean *z* Scores and Proportions With Stunting, Underweight, and Wasting LAZ indicates length for age *z* score; FCM, ferric carboxymaltose; SOC, standard of care; WAZ, weight-for-age *z* score; WLZ, weight-for-length *z* score.

Table 2 presents mean *z* scores at birth and at 1, 3, 6, 9, and 12 months. The distribution of raw *z* scores per visit is shown in the eFigure in Supplement 2. FCM did not have an impact on mean LAZ (mean difference, −0.15 [95% CI, −0.37 to 0.08]), WAZ (mean difference, −0.02 [95% CI, −0.21 to 0.16]), or WLZ (mean difference, 0.02 [95% CI, −0.20 to 0.24]) at any time point (Table 2 and Figure 2). Similar results were observed when *z* scores were corrected for gestational age at birth (eTable 2A in Supplement 2) or when excluding implausible values (eTable 2B in Supplement 2). Subgroup analyses with maternal baseline iron status, inflammation, placental malaria parasitemia, HIV status, age, height, education, and infant sex did not suggest heterogeneity in the treatment effects (eTable 3 in Supplement 2).

**Table 2.** *z* Scores by Treatment Group and Time Point

Outcome	FCM		SOC		Mean difference (95% CI)^b^	*P* value
	No. (%) (N = 371)^a^	Mean (SD)	No. (%) (N = 367)^a^	Mean (SD)		
LAZ						
Birth	357 (96.2)	−0.96 (1.71)	349 (95.1)	−0.92 (1.64)	−0.05 (−0.29 to 0.20)	.72
1 mo	252 (67.9)	−1.13 (2.03)	259 (70.6)	−1.14 (1.83)	0.04 (−0.29 to 0.37)	.82
3 mo	247 (66.6)	−1.02 (1.62)	254 (69.2)	−1.15 (1.67)	0.12 (−0.17 to 0.40)	.42
6 mo	285 (76.8)	−1.10 (1.41)	278 (75.7)	−1.19 (1.63)	0.12 (−0.13 to 0.37)	.33
9 mo	279 (75.2)	−1.27 (1.38)	264 (71.9)	−1.19 (1.49)	−0.01 (−0.25 to 0.22)	.91
12 mo	296 (79.8)	−1.40 (1.34)	274 (74.7)	−1.23 (1.50)	−0.15 (−0.37 to 0.08)	.21
WAZ						
Birth	370 (99.7)	−0.94 (1.14)	360 (98.1)	−0.90 (1.15)	−0.04 (−0.21 to 0.13)	.65
1 mo	252 (67.9)	−0.78 (1.28)	259 (70.6)	−0.62 (1.24)	−0.06 (−0.27 to 0.15)	.57
3 mo	247 (66.6)	−0.68 (1.32)	254 (69.2)	−0.60 (1.28)	−0.04 (−0.25 to 0.18)	.73
6 mo	285 (76.8)	−0.72 (1.29)	278 (75.7)	−0.66 (1.25)	0.05 (−0.15 to 0.25)	.63
9 mo	279 (75.2)	−0.97 (1.24)	264 (71.9)	−0.76 (1.26)	−0.10 (−0.30 to 0.09)	.30
12 mo	296 (79.8)	−0.88 (1.17)	274 (74.7)	−0.82 (1.24)	−0.02 (−0.21 to 0.16)	.79
WLZ						
Birth	301 (81.1)	−0.66 (1.86)	296 (80.7)	−0.59 (1.76)	−0.11 (−0.40 to 0.17)	.44
1 mo	241 (65.0)	0.03 (1.98)	251 (68.4)	0.37 (2.07)	−0.30 (−0.65 to 0.05)	.10
3 mo	246 (66.3)	0.24 (1.87)	251 (68.4)	0.44 (1.91)	−0.21 (−0.53 to 0.11)	.21
6 mo	285 (76.8)	0.09 (1.78)	276 (75.2)	0.21 (1.62)	−0.06 (−0.34 to 0.21)	.66
9 mo	279 (75.2)	−0.29 (1.53)	264 (71.9)	−0.04 (1.68)	−0.23 (−0.48 to 0.23)	.08
12 mo	296 (79.8)	−0.24 (1.43)	274 (74.7)	−0.27 (1.52)	0.02 (−0.20 to 0.24)	.87

Abbreviations: FCM, ferric carboxymaltose; LAZ, length-for-age *z* score; SOC, standard of care; WAZ, weight-for-age *z* score; WLZ, weight for length *z* score.

^a^ Includes all liveborn infants from the Randomized Controlled Trial of the Effect of Intravenous Iron on Anaemia in Malawian Pregnant Women (REVAMP) trial whose mothers consented to participate in the extended follow up and with at least 1 nonmissing outcome value. Of the 755 mothers who consented to their infants participating in the follow-up up to 12 months of age, 17 of 755 (2.3%) did not have data available at any 1 point in time.

^b^ Analyzed using a linear mixed model by Liang and Zeger,^30^ with a random intercept for participant and an unstructured variance-covariance among the repeated measurements. The fixed effects consisted of treatment, study visit, treatment × study visit interaction and site (the stratification factor). The models were fitted to all study visits from birth to 12 months of age. Results are presented as mean differences of FCM vs SOC with 2-sided 95% CIs with their associated *P* values.

Table 3 presents proportions with stunting, underweight, and wasting at birth, and at 1, 3, 6, 9, and 12 months. There was no evidence of significant differences in the proportion with stunting, underweight and wasting between the treatment groups at all time points. Growth faltering was present and notably high at birth, fluctuating only slightly over the 12 months (stunting, 177 of 706 [25.1%] at birth; 163 of 570 [28.6%] at 12 months; underweight, 102 of 730 [14.0%] at birth; 87 of 570 [15.3%] at 12 months), while wasting declined from 115 of 597 (19.3%) at birth to 71 of 570 (12.5%) at 12 months (Figure 2). Sensitivity analysis with *z* scores adjusted and corrected for gestational age, or excluding implausible values, showed similar results (eTables 2A and B in Supplement 2, respectively). The proportion with stunting was numerically higher at all time points among male infants, infants whose mothers were younger than 20 years, those whose mothers were shorter than 150 cm, and those whose mothers had no or primary education only, but there was no evidence of heterogeneity in intervention effects at all the time points (eTable 3 in Supplement 2).

**Table 3.** Stunting, Underweight, and Wasting by Treatment Group and Time Point

Outcome	FCM, No./ total No. (%)^a^	SOC, No./total No. (%)^a^	Risk ratio (95% CI)^b^	*P* value
Stunting, LAZ <−2				
Birth	93/357 (26.1)	84/349 (24.1)	1.10 (0.85 to 1.42)	.47
1 mo	64/252 (25.4)	65/259 (25.1)	1.01 (0.75 to 1.36)	.96
3 mo	50/247 (20.2)	65/254 (25.6)	0.78 (0.57 to 1.08)	.14
6 mo	62/285 (21.8)	66/278 (23.7)	0.89 (0.66 to 1.21)	.48
9 mo	78/279 (28.0)	73/264 (27.7)	0.98 (0.75 to 1.29)	.91
12 mo	90/296 (30.4)	73/274 (26.6)	1.13 (0.87 to 1.46)	.37
Underweight, WAZ <−2				
Birth	57/370 (15.4)	45/360 (12.5)	1.25 (0.86 to 1.83)	.25
1 mo	39/252 (15.5)	29/259 (11.2)	1.32 (0.84 to 2.06)	.23
3 mo	31/247 (12.6)	40/254 (15.8)	0.79 (0.51 to 1.22)	.28
6 mo	49/285 (17.2)	38/278 (13.7)	1.19 (0.80 to 1.76)	.39
9 mo	50/279 (17.9)	40/264 (15.2)	1.15 (0.79 to 1.67)	.47
12 mo	43/296 (14.5)	44/274 (16.1)	0.90 (0.62 to 1.32)	.60
Wasting, WLZ <−2				
Birth	62/301 (20.6)	53/296 (17.9)	1.20 (0.86 to 1.68)	.28
1 mo	32/241 (13.3)	27/251 (10.8)	1.23 (0.77 to 1.97)	.38
3 mo	22/246 (8.9)	23/251 (9.2)	1.02 (0.59 to 1.76)	.95
6 mo	33/285 (11.6)	22/276 (8.0)	1.44 (0.86 to 2.41)	.17
9 mo	33/279 (11.8)	21/264 (8.0)	1.51 (0.92 to 2.52)	.11
12 mo	32/296 (10.8)	39/274 (14.2)	0.78 (0.52 to 1.17)	.23

Abbreviations: FCM, ferric carboxymaltose; LAZ, length-for-age *z* score; SOC, standard of care; WAZ, weight-for-age *z* score; WLZ, weight for length *z* score.

^a^ Includes all liveborn infants from the Randomized Controlled Trial of the Effect of Intravenous Iron on Anaemia in Malawian Pregnant Women (REVAMP) trial whose mothers consented to participate in the extended follow up and with at least 1 nonmissing outcome value. Of the 755 mothers who consented to their infants participating in the follow-up up to 12 months of age, 17 of 755 (2.3%) did not have data available at any 1 point in time.

^b^ Analyzed using mixed effects Poisson regression models with a log link and robust SEs to directly estimate the risk ratio. The fixed effects consisted of treatment, study visit (categorical), treatment × study visit interaction, and site (the randomization stratification factor). These longitudinal models were fitted to all study visits from birth to 12 months of age. Results are presented as risk ratios of FCM vs SOC with 2-sided 95% CIs with their associated *P* values.

## Discussion

In the present study, we evaluated the effect of a single dose of IV FCM, compared with SOC oral iron, for the treatment of moderate to severe anemia during the second trimester of pregnancy, on child growth up to 12 months post partum, in Malawi, a resource-constrained setting in SSA. We observed that treatment with FCM had no substantial benefits over SOC on infant growth metrics over the first 12 postpartum months in the Malawian context.

Pregnancy interventions on child growth have increasingly been emphasized because of growing evidence that growth processes begin in early fetal life.^34,35^ Studies done more recently show that placental development and function are influenced by maternal nutritional status at conception and early pregnancy, and directly tie this to the child’s long-term health.^36,37^ Our study population includes pregnant women with moderate to severe anemia in the second trimester; probably, some women were already anemic at conception and in early pregnancy. Thus, the intervention timing may not have been optimal to improve infant outcomes. Other possible explanations for the observed limited effects could include the high baseline prevalence of malaria and inflammation in the study population.^27^ This indicates that the etiology of maternal anemia in this setting is highly multifactorial, involving a complex interplay between nutritional deficiencies and infection; thus, the effects of iron supplementation are likely to be less pronounced. Additionally, inflammation might have mitigated maternal metabolism and utilization of iron as well as restricted placental transfer of iron to the fetus, potentially limiting its effectiveness on maternal anemia and infant’s iron status, as observed in the REVAMP trial.^38^ These factors are on the pathway for infant growth.^37^

To our knowledge, our study is the first to report on both infant length and weight following maternal IV iron supplementation in pregnancy. Other studies examining IV iron in pregnancy have focused primarily on birth weight^27,39^ and weight gain^40^ only, overlooking the crucial role of length and stunting on human development. Systematic reviews of randomized clinical trials on IV vs oral iron for iron deficiency anemia in pregnancy have reported a benefit of IV iron on birth weight,^25,41^ although more recent trials in SSA have reported no effect.^27,39,42^ Thus, a better understanding of the role of pregnancy iron interventions on newborn and child growth in the SSA context is required.

Our findings are consistent with findings from observational studies evaluating oral iron in the SSA context. Two recent, large, pooled analyses using data from demographic and health surveys involving up to 23 countries in SSA showed no association between the use of prenatal oral iron supplementation and growth or stunting in children.^20,21^ This is similar to findings from a longitudinal observational birth cohort study in Cambridge, United Kingdom (the Cambridge Baby Growth Study), which reported no apparent differences in growth associated with maternal iron supplementation in pregnancy, despite an earlier reported positive effect on birthweight.^43^ This is, however, in contrast with observations from other LMICs in South Asia, which have reported significant improvement in postpartum growth and reduced risk of stunting following maternal oral iron supplementation during pregnancy.^18,19^ These differences highlight the importance of context on anemia and iron interventions. While iron deficiency is lower in high-income settings, unlike other LMICs, SSA faces additional challenges, such as higher burden of malaria and infectious disease-related inflammation. Thus, further studies to understand the role of prenatal iron on infant outcomes in both LMIC contexts are warranted.

Children in our cohort were on average lighter and shorter than the WHO’s standard growth reference values for length and weight.^44^ This was similar to children evaluated in Lungwena, in southern Malawi, a similar setting to where our study was conducted.^45,46,47^ The variation from the WHO standard reference could be the due to the effect of exposure to lean seasons—food availability is markedly seasonal in this region of Malawi—and the timing of this exposure as well as exposure to illnesses, as demonstrated by Maleta et al.^47,48^ Importantly, children in our cohort showed high rates of stunting present from birth, similar to those recruited in the Lungwena area.^46,49^ This also aligns with other studies, which have reported high rates of growth failure at birth in other low- and middle-income settings,^48,50^ suggesting that the contributing factors are established during the fetal period and thus underscoring the importance of preconception and prenatal interventions. Future investigations and interventions should ensure adequate maternal nutrition and health during pregnancy by targeting the preconception and early pregnancy periods, which hold greater potential for influencing growth trajectories.

Higher rates of stunting were observed among mothers younger than 20 years, with a height less than 150 cm, and with no or primary education only. Low levels of education and early parity have been associated with maternal anemia in pregnancy^51^ as well as growth faltering in children.^52^ It is probable that these characteristics are associated with other nutritional deficiencies alongside iron, which contribute to poor growth in infants; thus, while crucial, iron supplementation alone is less likely to be the optimal solution to improved infant growth outcomes. Moreover, previous studies in southern Malawi have shown that pregnant women with iron deficiency are likely to have other nutritional deficiencies, including protein and essential vitamins and minerals, such as vitamin D and zinc.^28,53^ Policy adjustments and interventions emphasizing dietary diversity, addressing early parity, and improving education and general socioeconomic support could be pivotal in mitigating growth failure in this population.

### Limitations

The study has limitations. The sample size was based on maternal anemia and neonatal birth weight outcomes, which might have limited the statistical power to detect small but meaningful differences in child growth over the 12 months. Importantly, although the overall data missingness was low (only 17 of 755 infants [2.3%] had no data at any time points), there was a higher level of data missingness present at each time point. This was mainly due to the COVID-19 pandemic and associated lockdowns, which affected participants’ attendance at the study visits, and delays in ethics approval to extend the trial follow-up. This, however, was comparable between the groups. Additionally, postnatal infant factors, known to be determinants of growth—including dietary diversity, other nutritional deficiencies, infectious disease, and mother-child interactions—were not evaluated and thus could not be accounted for. Further, even though the study drew from the rigorous randomization of the REVAMP trial, there could be some imbalances in unmeasured factors that could influence the results that were not accounted for in the analysis. Furthermore, while the trial setting represents a low-income setting, where nutrition deficiencies, malaria, infection, and inflammation coexist, which provides an ideal environment to unravel the benefits vs the risks of IV iron to determine its effect, it limits the generalizability of the findings to similar settings only.

## Conclusions

In this secondary analysis of a randomized clinical trial, there was no evidence of differences in growth between children whose mothers received IV FCM vs those who received SOC oral iron in the second trimester of pregnancy. While FCM in pregnant women with moderate to severely anemia improved maternal iron status compared with SOC, it showed limited efficacy in promoting infant growth in the Malawian context. Investigation and policies should aim at intervening in the preconception and early pregnancy periods, integrating maternal nutrition and infection control, and addressing early parity, education, and overall socioeconomic improvement to reduce growth faltering in similar settings.

## Notes

Supplement 1.
Trial Protocol and Statistical Analysis Plan

Supplement 2. **eFigure.** *z* Scores at Each Time Point **eTable 1.** Length and Weight by Maternal Treatment Group Across Visits, Main and Sensitivity Analyses **eTable 2.** *z* Scores and Stunting, Underweight, and Wasting by Treatment Group and Time Points: Sensitivity Analyses **eTable 3.** Treatment Effects on Growth and Maternal and Infant Characteristics: Subgroup Analyses

Supplement 3.
Data Sharing Statement
