# Supplementary material for: Prenatal Intravenous Iron and Child Growth: A Secondary Analysis of a Randomized Clinical Trial
Source: JAMA Netw Open. 2025 Oct 28;8(10):e2538392. doi: 10.1001/jamanetworkopen.2025.38392 (PMC12569709; doi:10.1001/jamanetworkopen.2025.38392)
Supplement: Supplement 2. — eFigure. z Scores at Each Time Point eTable 1. Length and Weight by Maternal Treatment Group Across Visits, Main and Sensitivity Analyses eTable 2. z Scores and Stunting, Underweight, and Wasting by Treatment Group and Time Points: Sensitivity Analyses eTable 3. Treatment Effects on Growth and Maternal and Infant Characteristics: Subgroup Analyses [file jamanetwopen-e2538392-s002.pdf]

Supplemental Online Content

Mzembe G, Nkhono W, Moya Moya E, et al. Prenatal intravenous iron and child growth: a secondary analysis of a randomized clinical trial. *JAMA Netw Open*. 2025;8(10):e2538392. doi:10.1001/jamanetworkopen.2025.38392

eFigure. *z* Scores at Each Time Point

eTable 1. Length and Weight by Maternal Treatment Group Across Visits, Main and Sensitivity Analyses

eTable 2. *z* Scores and Stunting, Underweight, and Wasting by Treatment Group and Time Points: Sensitivity Analyses

eTable 3. Treatment Effects on Growth and Maternal and Infant Characteristics: Subgroup Analyses

This supplemental material has been provided by the authors to give readers additional information about their work.

© 2025 Mzembe G et al. *JAMA Network Open*.

eFigure. *z* Scores at Each Time Point

The horizontal lines indicate the mean values; orange dots, FCM; blue dots, SOC.

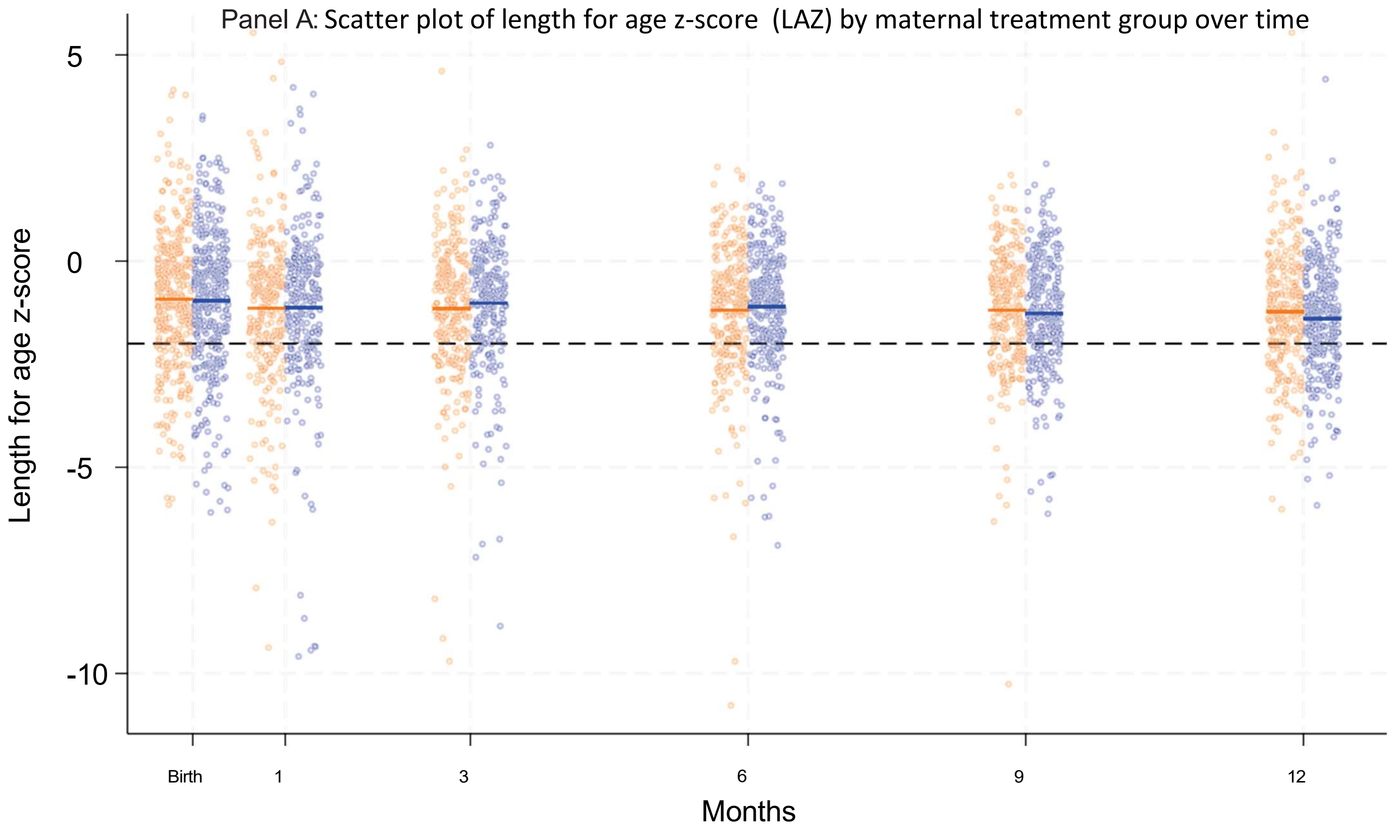

© 2025 Mzembe G et al. *JAMA Network Open*.

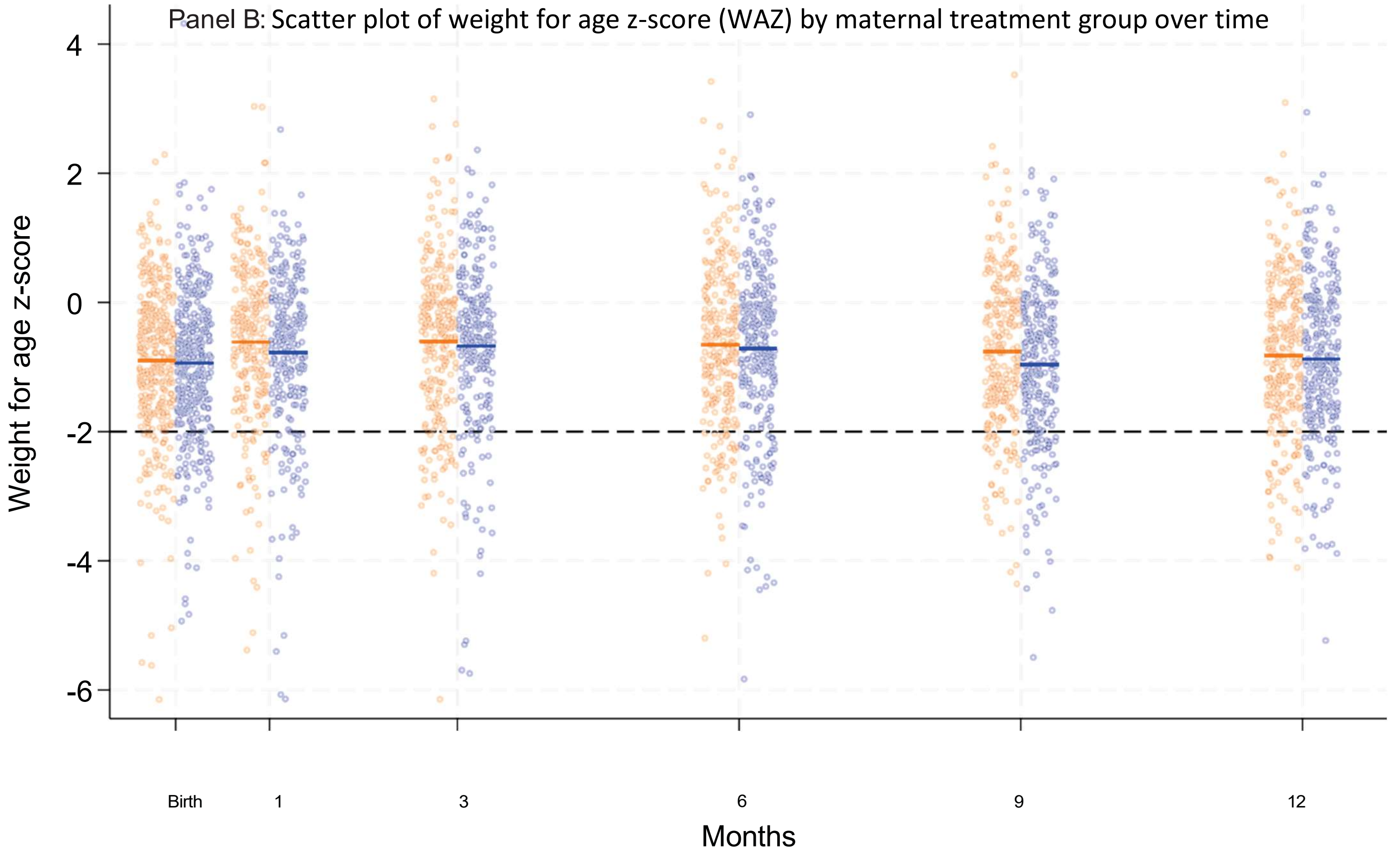

© 2025 Mzembe G et al. *JAMA Network Open*.

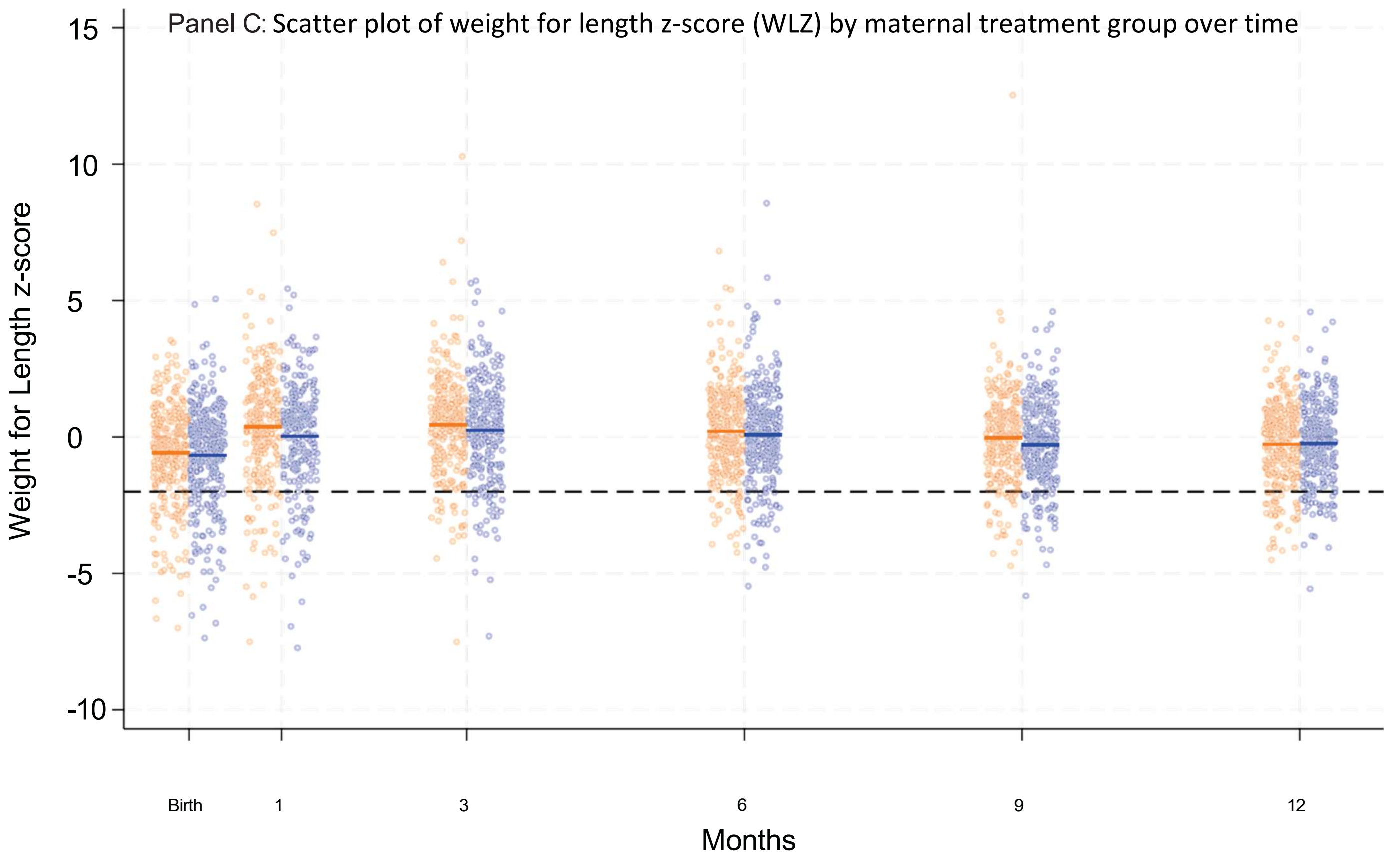

© 2025 Mzembe G et al. *JAMA Network Open*.

eTable 1. Length and Weight by Maternal Treatment Group Across Visits, Main and Sensitivity Analyses

eTable 1A. Length and weight by maternal treatment group across visits, main analysis

Outcome	Study visit	FCM		SOC		Mean difference[a] (95% CI)	Two-sided P value
		No. of infants N=371	Mean (SD)	No. of infants N=367[a]	Mean (SD)		
Length (cm)	Birth	357 (96.2)	47.7 (3.2)	349 (95.1)	47.8 (3.1)	-0.07 (-0.54, 0.39)	0.75
	1 month	252 (67.9)	52.0 (4.1)	261 (71.1)	51.9 (3.6)	0.10 (-0.55, 0.76)	0.75
	3 months	247 (66.6)	58.6 (3.5)	255 (69.5)	58.3 (3.5)	0.23 (-0.34, 0.86)	0.40
	6 months	285 (76.8)	64.3 (3.3)	280 (76.3)	64.1 (3.6)	0.29 (-0.27, 0.85)	0.31
	9 months	279 (75.2)	68.1 (3.1)	266 (72.5)	68.4 (3.5)	-0.04 (-0.59, 0.51)	0.89
	12 months	296 (79.8)	71.5 (3.3)	275 (74.9)	71.9 (3.6)	-0.36 (-0.92, 0.20)	0.20
Weight (g)	Birth	370 (99.7)	2892 (501)	362 (98.6)	2894 (516)	-3.03 (-76.69, 70.63)	0.94
	1 month	252 (67.9)	3913 (671)	261 (71.1)	3993 (680)	-13.85 (-127.20, 99.51)	0.81
	3 months	247 (66.6)	5706 (899)	255 (69.5)	5753 (926)	-15.41 (-165.05, 134.22)	0.84
	6 months	285 (76.8)	7091 (1042)	280 (76.3)	7136 (1075)	61.91 (-108.07, 231.90)	0.48
	9 months	279 (75.2)	7737 (1094)	266 (72.5)	7940 (1171)	-65.00 (-247.68, 117.69)	0.49
	12 months	296 (79.8)	8500 (1137)	275 (74.9)	8554 (1224)	-4.44 (-190.55, 181.66)	0.96

Abbreviations: FCM, Ferric carboxymaltose; SOC, Standard of care.

Includes all liveborn infants from the REVAMP trial whose mothers consented to participate in the extended follow up and with at least one non-missing outcome value. Of the mothers who consented to their infants participating in the follow-up up to 12 months of age, 17/755 (2.3%) did not have data available at any one point in time, thus, a total of 738 were included.

[a] Mean difference: An absolute mean difference of IV FCM versus SOC for length and weight at birth, 1, 3, 6, 9, and 12 months of age is shown following analyses using a longitudinal data analysis model (including all study visits from birth to 12 months) with a random intercept for participants and an unstructured variance-covariance among the repeated measurements.

eTable 2A (cont): *z* Scores and stunting, underweight and wasting by treatment group and time points: sensitivity analysis 1[a]

eTable 1B: Length and weight by maternal treatment group across visits: sensitivity analysis[a]

Outcome	Study visit	FCM		SOC		Mean difference[b] (95% CI)	Two-sided P value
		No of infants N=371	Mean (SD)	No of infants N=367	Mean (SD)		
Length (cm)	Birth	350 (94.3)	47.7 (3.1)	342 (93.2)	47.6 (2.9)	0.02 (-0.42, 0.47)	0.91
	1 month	240 (64.7)	52.3 (3.1)	250 (68.1)	52.0 (3.1)	0.31 (-0.23, 0.85)	0.26
	3 months	238 (64.2)	58.9 (2.9)	248 (67.6)	58.6 (3.0)	0.21 (-0.30, 0.72)	0.41
	6 months	281 (75.7)	64.4 (3.0)	275 (74.9)	64.3 (3.0)	0.22 (-0.27, 0.71)	0.38
	9 months	275 (74.1)	68.2 (3.0)	264 (71.9)	68.5 (3.1)	0.03 (-0.48, 0.54)	0.92
	12 months	295 (79.5)	71.5 (3.3)	275 (74.9)	71.9 (3.6)	-0.29 (-0.85, 0.27)	0.30
Weight (g)	Birth	364 (98.1)	2905 (491)	354 (96.5)	2902 (503)	-4.65 (-77.45, 68.15)	0.90
	1 month	245 (66.0)	3942 (622)	253 (68.9)	3982 (632)	1.20 (-105.38, 107.79)	0.98
	3 months	242 (65.2)	5721 (883)	250 (68.1)	5735 (854)	3.19 (-139.47, 145.86)	0.97
	6 months	283 (76.3)	7097 (1038)	278 (75.7)	7135 (1079)	73.58 (-96.06, 243.22)	0.40
	9 months	277 (74.7)	7760 (1062)	266 (72.5)	7940 (1171)	-57.79 (-238.42, 122.83)	0.53
	12 months	295 (79.5)	8511 (1122)	275 (74.9)	8554 (1224)	-3.33 (-187.84, 181.18)	0.97

Abbreviations: FCM, Ferric carboxymaltose; SOC, Standard of care.

[a] Implausible values, according to World Health Organization (WHO) standards for data exclusion, which include length-for-age z-scores (LAZ) below –6 or above +6, weight-for-age z-scores (WAZ) below –6 or above +5, and weight-for-length z-scores (WLZ) below –5 or above +5, were excluded from this analysis.

[b] Mean difference: An absolute mean difference of IV FCM versus SOC for length and weight at birth, 1, 3, 6, 9, and 12 months of age is shown following analyses using using a longitudinal data analysis model (including all study visits from birth to 12 months) with a random intercept for participants and an unstructured variance-covariance among the repeated measurements.

eTable 2. z Scores and Stunting, Underweight, and Wasting by Treatment Group and Time Points: Sensitivity Analyses

eTable 2A: *z* Scores and stunting, underweight and wasting by treatment group and time points: sensitivity analysis 1[a]

Outcome	Study visit	FCM		SOC		Mean difference[b] or Risk Ratio[c] (95% CI)	Two-sided P value
		No of infants N=371	Mean (SD) or n/N (%)	No of infants N=367	Mean (SD) or n/N (%)		
**Length-for-age**							
Length-for-age z-scores (LAZ)	Birth	353 (95.1)	-0.81 (1.65)	343 (93.5)	-0.75 (1.58)	-0.05 (-0.28, 0.19)	0.71
	1 month	252 (67.9)	-0.95 (1.82)	259 (70.6)	-0.98 (1.74)	0.04 (-0.26, 0.35)	0.79
	3 months	247 (66.6)	-0.88 (1.51)	254 (69.2)	-1.03 (1.69)	0.12 (-0.16, 0.40)	0.41
	6 months	285 (76.8)	-1.03 (1.36)	277 (75.5)	-1.13 (1.62)	0.10 (-0.14, 0.35)	0.41
	9 months	257 (69.3)	-1.21 (1.35)	244 (66.5)	-1.13 (1.50)	-0.04 (-0.29, 0.20)	0.73
	12 months	274 (73.8)	-1.36 (1.31)	254 (69.2)	-1.16 (1.51)	-0.21 (-0.44, 0.03)	0.09
Stunting (LAZ<-2)	Birth	353 (95.1)	85/353 (24.1)	343 (93.5)	73/343 (21.3)	1.15 (0.87, 1.51)	0.34
	1 month	252 (67.9)	55/252 (21.8)	259 (70.6)	56/259 (21.6)	1.01 (0.73, 1.40)	0.95
	3 months	247 (66.6)	45/247 (18.2)	254 (69.2)	59/254 (23.2)	0.78 (0.55, 1.11)	0.17
	6 months	285 (76.8)	57/285 (20.0)	277 (75.5)	61/277 (22.0)	0.90 (0.66, 1.24)	0.53
	9 months	257 (69.3)	66/257 (25.7)	244 (66.5)	61/244 (25.0)	1.03 (0.76, 1.38)	0.87
	12 months	274 (73.8)	80/274 (29.2)	254 (69.2)	62/254 (24.4)	1.20 (0.90, 1.60)	0.21
**Weight- for-age**							
Weight- for-age z-score (WAZ)	Birth	366 (98.7)	-0.85 (1.00)	356 (97.0)	-0.79 (0.99)	-0.07 (-0.22, 0.08)	0.36
	1 month	252 (67.9)	-0.61 (1.12)	259 (70.6)	-0.47 (1.13)	-0.11 (-0.30, 0.08)	0.26
	3 months	247 (66.6)	-0.55 (1.25)	254 (69.2)	-0.49 (1.25)	-0.07 (-0.27, 0.14)	0.53
	6 months	285 (76.8)	-0.67 (1.25)	277 (75.5)	-0.62 (1.24)	0.01 (-0.19, 0.21)	0.91
	9 months	257 (69.3)	-0.96 (1.23)	244 (66.5)	-0.71 (1.27)	-0.16 (-0.36, 0.04)	0.11
	12 months	274 (73.8)	-0.86 (1.15)	254 (69.2)	-0.75 (1.23)	-0.08 (-0.27, 0.10)	0.38
Underweight (WAZ<-2)	Birth	366 (98.7)	40/366 (10.9)	356 (97.0)	42/356 (11.8)	0.94 (0.62, 1.44)	0.79
	1 month	252 (67.9)	31/252 (12.3)	259 (70.6)	22/259 (8.5)	1.42 (0.85, 2.38)	0.18
	3 months	247 (66.6)	27/247 (10.9)	254 (69.2)	34/254 (13.4)	0.82 (0.51, 1.32)	0.42
	6 months	285 (76.8)	44/285 (15.4)	277 (75.5)	34/277 (12.3)	1.22 (0.80, 1.84)	0.36
	9 months	257 (69.3)	44/257 (17.1)	244 (66.5)	37/244 (15.2)	1.10 (0.75, 1.63)	0.62

eTable 2A (cont): *z* Scores and stunting, underweight and wasting by treatment group and time points: sensitivity analysis 1[a]

Outcome	Study visit	FCM		SOC		Mean difference[b] or Risk Ratio[c] (95% CI)	Two-sided P value
		No of infants N=371	Mean (SD) or n/N (%)	No of infants N=367	Mean (SD) or n/N (%)		
	12 months	274 (73.8)	37/274 (13.5)	254 (69.2)	37/254 (14.6)	0.92 (0.62, 1.40)	0.73
**Weight-for-length**							
Weight-for-length z-score (WLZ)	Birth	318 (85.7)	-0.55 (1.85)	308 (83.9)	-0.55 (1.74)	-0.03 (-0.31, 0.25)	0.82
	1 month	248 (66.8)	0.12 (2.05)	254 (69.2)	0.41 (2.17)	-0.24 (-0.61, 0.12)	0.19
	3 months	246 (66.3)	0.31 (1.93)	250 (68.8)	0.53 (1.90)	-0.22 (-0.54, 0.11)	0.19
	6 months	281 (75.7)	0.20 (1.88)	273 (74.4)	0.33 (1.72)	-0.07 (-0.36, 0.22)	0.65
	9 months	264 (71.2)	-0.26 (1.62)	249 (67.8)	0.02 (1.73)	-0.24 (-0.52, 0.03)	0.08
	12 months	276 (74.4)	-0.22 (1.43)	254 (69.2)	-0.23 (1.54)	0.03 (-0.21, 0.26)	0.81
Wasting (WLZ<-2)	Birth	318 (85.7)	61/318 (20.1)	308 (83.9)	52/308 (16.9)	1.23 (0.88, 1.72)	0.22
	1 month	248 (66.8)	32/248 (12.9)	254 (69.2)	25/254 (9.8)	1.30 (0.81, 2.11)	0.28
	3 months	246 (66.3)	22/246 (8.9)	250 (68.8)	23/250 (9.2)	1.01 (0.59, 1.75)	0.96
	6 months	281 (75.7)	31/281 (11.0)	273 (74.4)	22/273 (8.1)	1.36 (0.80, 2.28)	0.24
	9 months	264 (71.2)	32/264 (12.1)	249 (67.8)	21/249 (8.4)	1.43 (0.86, 2.38)	0.17
	12 months	276 (74.4)	30/276 (10.9)	254 (69.2)	36/254 (14.2)	0.77 (0.51, 1.19)	0.25

Abbreviations: FCM, Ferric carboxymaltose; SOC, Standard of care.

Includes all liveborn infants from the REVAMP trial whose mothers consented to participate in the extended follow up and with at least one non-missing outcome value. Of the mothers who consented to their infants participating in the follow-up up to 12 months of age, 17/755 (2.3%) did not have data available at any one point in time, thus, a total of 738 were included.

[a] Underlying z-scores were derived using the INTERGROWTH-21st's gigs package which adjusts for a combination of gestation age and postnatal age.

[b] Mean difference: An absolute mean difference of FCM versus SOC for length-for-age z-scores (LAZ), weight-for-age z-scores (WAZ), and weight-for-length z-scores (WLZ) at birth, 1, 3, 6, 9, and 12 months of age is shown following analyses using a longitudinal data analysis model (including all study visits from birth to 12 months) with a random intercept for participants and an unstructured variance-covariance among the repeated measurements.

[c] Risk ratio: A risk ratio of FCM versus SOC for stunting, wasting and underweight at birth, 1, 3, 6, 9, and 12 months of age is shown following analyses using a Poisson model with random intercept for the infants and robust standard errors.

eTable 2B: *z* Scores and stunting, underweight and wasting by treatment group and time points: sensitivity analysis 2[a]

Outcome	Study visit	FCM		SOC		Mean difference[b] or Risk Ratio[c] (95% CI)	Two-sided P value
		No of infants N=371	Mean (SD) or n/N (%)	No of infants N=367	Mean (SD) or n/N (%)		
Length-for-age							
Length-for-age z-scores (LAZ)	Birth	356 (96.0)	-0.95 (1.69)	349 (95.1)	-0.92 (1.64)	-0.03 (-0.28, 0.21)	0.81
	1 month	245 (66.0)	-0.91 (1.60)	256 (69.8)	-1.06 (1.68)	0.14 (-0.14, 0.43)	0.32
	3 months	243 (65.5)	-0.92 (1.42)	251 (68.4)	-1.05 (1.43)	0.09 (-0.16, 0.33)	0.50
	6 months	283 (76.3)	-1.07 (1.34)	275 (74.9)	-1.10 (1.40)	0.10 (-0.12, 0.32)	0.38
	9 months	277 (74.7)	-1.23 (1.31)	262 (71.4)	-1.13 (1.36)	-0.00 (-0.23, 0.22)	0.97
	12 months	296 (79.8)	-1.40 (1.34)	274 (74.7)	-1.23 (1.50)	-0.13 (-0.35, 0.10)	0.28
Stunting (LAZ<-2)	Birth	356 (96.0)	92/356 (25.8)	349 (95.1)	84/349 (24.1)	1.09 (0.84-1.42)	0.51
	1 month	245 (66.0)	57/245 (23.3)	256 (69.8)	62/256 (24.2)	0.97 (0.71-1.32)	0.82
	3 months	243 (65.5)	46/243 (18.9)	251 (68.4)	62/251 (24.7)	0.77 (0.55-1.07)	0.12
	6 months	283 (76.3)	60/283 (21.2)	275 (74.9)	63/275 (22.9)	0.90 (0.66-1.23)	0.51
	9 months	277 (74.7)	76/277 (27.4)	262 (71.4)	71/262 (27.1)	0.98 (0.75-1.30)	0.91
	12 months	296 (79.8)	90/296 (30.4)	274 (74.7)	73/274 (26.6)	1.13 (0.87-1.47)	0.37
Weight- for-age							
Weight- for-age z-score (WAZ)	Birth	370 (99.7)	-0.94 (1.14)	359 (97.8)	-0.89 (1.12)	-0.05 (-0.22, 0.11)	0.53
	1 month	250 (67.4)	-0.73 (1.20)	259 (70.6)	-0.62 (1.24)	-0.06 (-0.26, 0.15)	0.57
	3 months	247 (66.6)	-0.68 (1.32)	253 (68.9)	-0.58 (1.24)	-0.07 (-0.28, 0.14)	0.52
	6 months	285 (76.8)	-0.72 (1.29)	278 (75.7)	-0.66 (1.25)	0.05 (-0.16, 0.25)	0.65
	9 months	279 (75.2)	-0.97 (1.24)	264 (71.9)	-0.76 (1.26)	-0.11 (-0.30, 0.09)	0.29
	12 months	296 (79.8)	-0.88 (1.17)	274 (74.7)	-0.82 (1.24)	-0.03 (-0.21, 0.16)	0.77
Underweight (WAZ<-2)	Birth	370 (99.7)	57/370 (15.4)	359 (97.8)	44/359 (12.3)	1.28 (0.87-1.88)	0.20
	1 month	250 (67.4)	37/250 (14.8)	259 (70.6)	29/259 (11.2)	1.30 (0.82-2.04)	0.26
	3 months	247 (66.6)	31/247 (12.6)	253 (68.9)	39/253 (15.4)	0.81 (0.53-1.26)	0.35
	6 months	285 (76.8)	49/285 (17.2)	278 (75.7)	38/278 (13.7)	1.19 (0.81-1.77)	0.38
	9 months	279 (75.2)	50/279 (17.9)	264 (71.9)	40/264 (15.2)	1.15 (0.79-1.68)	0.46
	12 months	296 (79.8)	43/296 (14.5)	274 (74.7)	44/274 (16.1)	0.91 (0.62-1.33)	0.62
Weight-for-length							
Weight-for-length z-score (WLZ)	Birth	295 (79.5)	-0.55 (1.70)	289 (78.7)	-0.46 (1.57)	-0.13 (-0.39, 0.14)	0.35
	1 month	236 (63.6)	0.12 (1.77)	243 (66.2)	0.37 (1.74)	-0.23 (-0.54, 0.08)	0.14
	3 months	238 (64.2)	0.23 (1.69)	246 (67.0)	0.36 (1.61)	-0.17 (-0.45, 0.11)	0.23

eTable 2B (cont): ***z*** **Scores and stunting, underweight and wasting by treatment group and time points: sensitivity analysis 2**[a]

Outcome	Study visit	FCM		SOC		Mean difference[b] or Risk Ratio[c] (95% CI)	Two-sided P value
		No of infants N=371	Mean (SD) or n/N (%)	No of infants N=367	Mean (SD) or n/N (%)		
	6 months	281 (75.7)	0.05 (1.64)	273 (74.4)	0.15 (1.52)	-0.05 (-0.30, 0.21)	0.70
	9 months	275 (74.1)	-0.28 (1.45)	262 (71.4)	-0.09 (1.48)	-0.21 (-0.44, 0.02)	0.07
	12 months	295 (79.5)	-0.22 (1.40)	274 (74.7)	-0.27 (1.52)	0.02 (-0.20, 0.24)	0.86
Wasting (WLZ<-2)	Birth	295 (79.5)	56/295 (19.0)	289 (78.7)	46/289 (15.9)	1.25 (0.88-1.79)	0.22
	1 month	236 (63.6)	28/236 (11.9)	243 (66.2)	23/243 (9.5)	1.25 (0.75-2.07)	0.40
	3 months	238 (64.2)	20/238 (8.4)	246 (67.0)	22/246 (8.9)	0.98 (0.56-1.73)	0.95
	6 months	281 (75.7)	32/281 (11.4)	273 (74.4)	22/273 (8.1)	1.40 (0.83-2.35)	0.20
	9 months	275 (74.1)	31/275 (11.3)	262 (71.4)	21/262 (8.0)	1.45 (0.87-2.42)	0.15
	12 months	295 (79.5)	31/295 (10.5)	274 (74.7)	39/274 (14.2)	0.76 (0.50-1.16)	0.20

Abbreviations: FCM, Ferric carboxymaltose; SOC, Standard of care.

[a] Implausible values according to World Health Organization (WHO) standards for data exclusion, which include length-for-age z-scores (LAZ) below –6 or above +6, weight-for-age z-scores (WAZ) below –6 or above +5, and weight-for-length z-scores (WLZ) below –5 or above +5, were excluded from this analysis.

[b] Mean difference: An absolute mean difference of FCM versus SOC for LAZ, WAZ, and WLZ at birth, 1, 3, 6, 9, and 12 months of age is shown following analyses using a longitudinal data analysis model (including all study visits from birth to 12 months) with a random intercept for participant and an unstructured variance-covariance among the repeated measurements.

[c] Risk ratio: A risk ratio of FCM versus SOC stunting, wasting and underweight at birth, 1, 3, 6, 9, and 12 months of age is shown following analyses using mixed effects Poisson regression models with a log link and robust standard errors.

© 2025 Mzembe G et al. *JAMA Network Open*.

eTable 3: Treatment effects on Growth and Maternal and Infant Characteristics: Subgroup analyses

	Overall Mean (SD) or n/N (%)	FCM Mean (SD) or n/N (%)	SOC Mean (SD)/ n/N (%)	Mean Difference[a] or Risk Ratio[b] (95% CI)	Two-sided P-value	Overall Mean (SD) or n/N (%)	FCM Mean (SD) or n/N (%)	SOC Mean (SD) or n/N (%)	Mean Difference[a] or Risk Ratio[b] (95% CI)	Two-sided P-value	Inter-action P-value
**LENGTH-FOR-AGE Z-SCORES (LAZ)**											
Iron status at randomization											
	Iron deficient					Non-deficient					
Birth	-0.73 (1.67)	-0.87 (1.77)	-0.59 (1.56)	-0.28 (-0.66, 0.10)	0.15	-1.07 (1.67)	-1.00 (1.68)	-1.13 (1.66)	0.11 (-0.22, 0.44)	0.51	0.13
1 month	-0.75 (1.82)	-0.67 (1.79)	-0.83 (1.85)	0.16 (-0.36, 0.68)	0.54	-1.39 (1.98)	-1.47 (2.18)	-1.31 (1.78)	-0.12 (-0.56, 0.31)	0.58	0.41
3 months	-0.87 (1.65)	-0.76 (1.58)	-0.99 (1.71)	0.19 (-0.26, 0.64)	0.41	-1.25 (1.65)	-1.22 (1.67)	-1.28 (1.63)	0.08 (-0.29, 0.46)	0.67	0.72
6 months	-0.91 (1.30)	-0.88 (1.26)	-0.95 (1.34)	0.07 (-0.32, 0.46)	0.71	-1.31 (1.65)	-1.27 (1.51)	-1.35 (1.79)	0.12 (-0.20, 0.45)	0.46	0.85
9 months	-1.07 (1.30)	-1.05 (1.26)	-1.10 (1.35)	0.07 (-0.29, 0.44)	0.69	-1.33 (1.53)	-1.41 (1.46)	-1.26 (1.59)	-0.08 (-0.39, 0.24)	0.64	0.54
12 months	-1.24 (1.40)	-1.34 (1.24)	-1.14 (1.53)	-0.21 (-0.56, 0.15)	0.26	-1.35 (1.45)	-1.41 (1.43)	-1.28 (1.47)	-0.10 (-0.41, 0.21)	0.53	0.65
Inflammation at randomization											
	Yes					No					
Birth	-0.94 (1.72)	-1.05 (1.80)	-0.83 (1.63)	-0.22 (-0.57, 0.12)	0.20	-0.91 (1.63)	-0.82 (1.61)	-1.00 (1.65)	0.15 (-0.20, 0.51)	0.40	0.14
1 month	-1.01 (1.86)	-1.06 (2.08)	-0.95 (1.62)	-0.10 (-0.57, 0.37)	0.68	-1.24 (2.01)	-1.21 (2.05)	-1.29 (1.99)	0.12 (-0.35, 0.61)	0.60	0.50
3 months	-1.08 (1.61)	-0.88 (1.50)	-1.28 (1.68)	0.49 (0.09, 0.90)	0.02	-1.11 (1.71)	-1.18 (1.78)	-1.04 (1.65)	-0.25 (-0.66, 0.15)	0.23	0.01
6 months	-1.13 (1.31)	-1.02 (1.27)	-1.23 (1.34)	0.22 (-0.12, 0.58)	0.21	-1.16 (1.73)	-1.20 (1.57)	-1.13 (1.88)	-0.01 (-0.37, 0.35)	0.95	0.35
9 months	-1.29 (1.42)	-1.29 (1.36)	-1.28 (1.49)	0.08 (-0.26, 0.42)	0.63	-1.16 (1.46)	-1.22 (1.42)	-1.10 (1.50)	-0.09 (-0.44, 0.25)	0.59	0.47
12 months	-1.36 (1.42)	-1.41 (1.34)	-1.31 (1.51)	-0.05 (-0.38, 0.28)	0.76	-1.24 (1.43)	-1.35 (1.36)	-1.13 (1.49)	-0.22 (-0.55, 0.10)	0.18	0.47
Placental malaria											
	Positive					Negative					
Birth	-1.18 (1.57)	-1.20 (1.48)	-1.16 (1.65)	-0.02 (-0.46, 0.42)	0.92	-0.86 (1.75)	-0.85 (1.73)	-0.86 (1.78)	-0.01 (-0.36, 0.32)	0.91	0.18
1 month	-1.22 (1.80)	-1.24 (2.02)	-1.19 (1.60)	-0.02 (-0.62, 0.58)	0.95	-1.09 (2.12)	-0.60 (2.06)	-1.04 (2.19)	0.15 (-0.32, 0.63)	0.51	0.45
3 months	-1.05 (1.51)	-1.12 (1.56)	-0.97 (1.47)	-0.17 (-0.68, 0.34)	0.51	-1.17 (1.81)	-1.40 (1.87)	-0.94 (1.74)	0.46 (0.07, 0.87)	0.02	0.94
6 months	-1.16 (1.52)	-1.17 (1.49)	-1.15 (1.56)	0.01 (-0.45, 0.47)	0.96	-1.15 (1.60)	-1.28 (1.72)	-1.03 (1.47)	0.28 (-0.07, 0.63)	0.11	0.52
9 months	-1.12 (1.34)	-1.30 (1.43)	-0.93 (1.24)	-0.26 (-0.69, 0.17)	0.23	-1.29 (1.51)	-1.32 (1.66)	-1.25 (1.37)	0.11 (-0.22, 0.43)	0.53	0.94
12 months	-1.25 (1.33)	-1.44 (1.32)	-1.04 (1.31)	-0.34 (-0.75, 0.08)	0.11	-1.36 (1.48)	-1.32 (1.58)	-1.38 (1.38)	-0.05 (-0.36, 0.27)	0.77	0.52
Maternal HIV status											
	Positive					Negative					
Birth	-0.69 (1.54)	-0.66 (1.67)	-0.72 (1.41)	0.04 (-0.56, 0.63)	0.91	-0.99 (1.70)	-1.04 (1.71)	-0.95 (1.68)	-0.09 (-0.36, 0.18)	0.52	0.71
1 month	-0.82 (2.00)	-0.70 (1.56)	-0.97 (2.45)	0.07 (-0.76, 0.91)	0.86	-1.20 (1.92)	-1.22 (2.12)	-1.17 (1.72)	0.01 (-0.35, 0.37)	0.96	0.90
3 months	-0.84 (1.65)	-0.90 (1.75)	-0.77 (1.55)	-0.28 (-0.99, 0.42)	0.43	-1.15 (1.64)	-1.06 (1.59)	-1.23 (1.69)	0.19 (-0.12, 0.50)	0.23	0.22
6 months	-1.17 (1.48)	-1.20 (1.33)	-1.13 (1.65)	-0.22 (-0.82, 0.38)	0.47	-1.15 (1.53)	-1.08 (1.43)	-1.22 (1.62)	0.21 (-0.06, 0.49)	0.13	0.20
9 months	-1.28 (1.56)	-1.40 (1.49)	-1.14 (1.64)	-0.24 (-0.81, 0.32)	0.40	-1.22 (1.40)	-1.23 (1.35)	-1.22 (1.46)	0.06 (-0.21, 0.32)	0.68	0.35
12 months	-1.42 (1.36)	-1.36 (1.36)	-1.47 (1.37)	0.02 (-0.53, 0.56)	0.96	-1.30 (1.43)	-1.40 (1.35)	-1.20 (1.52)	-0.16 (-0.41, 0.10)	0.22	0.57
Maternal age											
	Age <20 years					Age ≥20 years					
Birth	-1.11 (1.61)	-1.16 (1.62)	-1.06 (1.61)	-0.11 (-0.48, 0.26)	0.55	-0.81 (1.72)	-0.81 (1.77)	-0.81 (1.67)	0.00 (-0.33, 0.33)	1.00	0.66
1 month	-1.42 (1.92)	-1.56 (2.17)	-1.30 (1.67)	-0.25 (-0.73, 0.23)	0.30	-0.87 (1.91)	-0.77 (1.85)	-0.98 (1.97)	0.27 (-0.19, 0.72)	0.25	0.12
3 months	-1.37 (1.67)	-1.30 (1.67)	-1.45 (1.67)	0.18 (-0.23, 0.58)	0.39	-1.80 (1.58)	-0.77 (1.54)	-0.85 (1.63)	0.03 (-0.35, 0.42)	0.86	0.62
6 months	-1.38 (1.64)	-1.30 (1.64)	-1.47 (1.64)	0.23 (-0.13, 0.60)	0.21	-0.95 (1.39)	-0.95 (1.17)	-0.94 (1.59)	0.01 (-0.32, 0.34)	0.96	0.38
9 months	-1.41 (1.49)	-1.43 (1.45)	-1.39 (1.53)	0.04 (-0.30, 0.39)	0.81	-1.07 (1.36)	-1.13 (1.30)	-0.99 (1.43)	-0.08 (-0.40, 0.24)	0.62	0.60
12 months	-1.49 (1.41)	-1.58 (1.39)	-1.40 (1.43)	-0.14 (-0.48, 0.19)	0.41	-1.16 (1.42)	-1.24 (1.29)	-1.08 (1.54)	-0.16 (-0.47, 0.15)	0.30	0.93
Maternal height											
	Height <150 cm					Height ≥150 cm					
Birth	-1.29 (1.73)	-1.09 (1.69)	-1.47 (1.75)	0.37 (-0.23, 0.96)	0.22	-0.87 (1.66)	-0.94 (1.72)	-0.80 (1.60)	-0.14 (-0.41, 0.13)	0.29	0.12
1 month	-1.56 (2.03)	-1.78 (2.45)	-1.34 (1.50)	-0.35 (-1.13, 0.43)	0.38	-1.04 (1.90)	-0.98 (1.91)	-1.10 (1.89)	0.12 (-0.24, 0.48)	0.51	0.28

eTable 3 (cont): Treatment effects on Growth and Maternal and Infant Characteristics: Subgroup analyses

	Overall Mean (SD) or n/N (%)	FCM Mean (SD) or n/N (%)	SOC Mean (SD)/ n/N (%)	Mean Difference[a] or Risk Ratio[b] (95% CI)	Two-sided P-value	Overall Mean (SD) or n/N (%)	FCM Mean (SD) or n/N (%)	SOC Mean (SD) or n/N (%)	Mean Difference[a] or Risk Ratio[b] (95% CI)	Two-sided P-value	Inter-action P-value
3 months	-1.66 (1.76)	-1.66 (1.82)	-1.67 (1.73)	0.22 (-0.45, 0.90)	0.51	-0.96 (1.60)	-0.89 (1.55)	-1.03 (1.64)	0.09 (-0.22, 0.40)	0.58	0.72
6 months	-1.64 (1.48)	-1.61 (1.38)	-1.67 (1.58)	0.16 (-0.44, 0.75)	0.61	-1.04 (1.51)	-1.00 (1.39)	-1.09 (1.63)	0.11 (-0.16, 0.38)	0.43	0.89
9 months	-1.62 (1.18)	-1.64 (1.06)	-1.61 (1.30)	0.11 (-0.45, 0.67)	0.70	-1.14 (1.47)	-1.19 (1.42)	-1.09 (1.52)	-0.05 (-0.31, 0.21)	0.70	0.60
12 months	-1.66 (1.46)	-1.82 (1.19)	-1.49 (1.71)	-0.20 (-0.75, 0.34)	0.46	-1.24 (1.40)	-1.31 (1.36)	-1.17 (1.45)	-0.14 (-0.39, 0.11)	0.27	0.84
Maternal education											
	None/Primary					Secondary/ Tertiary					
Birth	-1.10 (1.67)	-1.09 (1.63)	-1.12 (1.72)	0.02 (-0.29, 0.34)	0.89	-0.67 (1.68)	-0.73 (1.86)	-0.61 (1.47)	-0.12 (-0.54, 0.29)	0.56	0.59
1 month	-1.30 (1.98)	-1.24 (2.15)	-1.37 (1.80)	0.12 (-0.29, 0.54)	0.56	-0.90 (1.82)	-0.99 (1.84)	-0.83 (1.81)	-0.10 (-0.67, 0.48)	0.74	0.54
3 months	-1.17 (1.66)	-1.13 (1.54)	-1.21 (1.78)	0.05 (-0.31, 0.40)	0.79	-0.88 (1.62)	-0.76 (1.74)	-0.99 (1.50)	0.25 (-0.24, 0.75)	0.32	0.51
6 months	-1.25 (1.52)	-1.24 (1.47)	-1.26 (1.57)	0.07 (-0.25, 0.38)	0.68	-0.93 (1.49)	-0.85 (1.29)	-1.00 (1.66)	0.17 (-0.25, 0.59)	0.43	0.70
9 months	-1.38 (1.47)	-1.39 (1.41)	-1.36 (1.53)	-0.02 (-0.31, 0.28)	0.92	-0.95 (1.30)	-1.02 (1.28)	-0.89 (1.32)	-0.03 (-0.43, 0.37)	0.88	0.95
12 months	-1.44 (1.41)	-1.53 (1.38)	-1.34 (1.45)	-0.16 (-0.45, 0.12)	0.26	-1.13 (1.32)	-1.19 (1.24)	-1.06 (1.39)	-0.09 (-0.47, 0.29)	0.64	0.76
Infant sex											
	Female					Male					
Birth	-0.79 (1.53)	-0.76 (1.60)	-0.82 (1.47)	0.06 (-0.30, 0.41)	0.75	-1.08 (1.79)	-1.15 (17.9)	-1.02 (1.80)	-0.13 (-0.48, 0.21)	0.44	0.44
1 month	-1.07 (1.83)	-1.10 (1.86)	-1.04 (1.81)	-0.09 (-0.56, 0.37)	0.70	-1.20 (2.03)	-1.15 (2.20)	-1.25 (1.85)	0.18 (-0.29, 0.64)	0.46	0.43
3 months	-0.99 (1.53)	-0.95 (1.60)	-1.03 (1.48)	0.02 (-0.38, 0.43)	0.91	-1.17 (1.75)	-1.08 (1.65)	-1.27 (1.85)	0.21 (-0.18, 0.61)	0.30	0.51
6 months	-0.99 (1.54)	-1.03 (1.47)	-0.96 (1.61)	-0.05 (-0.40, 0.30)	0.79	-1.30 (1.49)	-1.17 (1.35)	-1.43 (1.63)	0.30 (-0.05, 0.65)	0.09	0.17
9 months	-0.98 (1.41)	-0.98 (1.27)	-0.98 (1.55)	-0.02 (-0.36, 0.31)	0.90	-1.47 (1.42)	-1.56 (1.42)	-1.39 (1.41)	-0.01 (-0.34, 0.32)	0.96	0.95
12 months	-1.07 (1.34)	-1.23 (1.16)	-0.92 (1.49)	-0.32 (-0.64, 0.00)	0.05	-1.54 (1.46)	-1.56 (1.48)	-1.54 (1.44)	0.03 (-0.28, 0.35)	0.83	0.12
**WEIGHT-FOR-AGE Z-SCORES (WAZ)**											
Iron status at randomization											
	Iron deficient					Non-deficient					
Birth	-0.74 (1.07)	-0.75 (1.07)	-0.73 (1.06)	-0.01 (-0.28, 0.25)	0.91	-1.05 (1.19)	-1.08 (1.19)	-1.02 (1.20)	-0.07 (-0.29, 0.15)	0.53	0.74
1 month	-0.49 (1.17)	-0.62 (1.20)	-0.36 (1.14)	-0.13 (-0.46, 0.20)	0.45	-0.84 (1.31)	-0.91 (1.34)	-0.78 (1.28)	-0.04 (-0.32, 0.23)	0.75	0.70
3 months	-0.50 (1.27)	-0.62 (1.27)	-0.38 (1.28)	-0.22 (-0.56, 0.12)	0.20	-0.75 (1.31)	-0.73 (1.37)	-0.77 (1.26)	0.09 (-0.19, 0.37)	0.54	0.17
6 months	-0.62 (1.25)	-0.75 (1.29)	-0.51 (1.21)	-0.15 (-0.46, 0.17)	0.36	-0.74 (1.26)	-0.71 (1.29)	-0.78 (1.24)	0.19 (-0.08, 0.46)	0.16	0.11
9 months	-0.85 (1.26)	-1.00 (1.26)	-0.70 (1.26)	-0.27 (-0.58, 0.03)	0.08	-0.88 (1.25)	-0.94 (1.24)	-0.82 (1.25)	0.03 (-0.23, 0.29)	0.84	0.14
12 months	-0.85 (1.25)	-0.87 (1.24)	-0.85 (1.27)	0.01 (-0.27, 0.30)	0.93	-0.85 (1.17)	-0.88 (1.13)	-0.82 (1.21)	-0.04 (-0.28, 0.21)	0.76	0.79
Inflammation at randomization											
	Yes					No					
Birth	-0.99 (1.18)	-1.08 (1.15)	-0.89 (1.20)	-0.20 (-0.44, 0.03)	0.09	-0.85 (1.11)	-0.79 (1.13)	-0.92 (1.10)	0.14 (-0.10, 0.39)	0.26	0.05
1 month	-0.71 (1.22)	-0.81 (1.23)	-0.62 (1.21)	-0.15 (-0.45, 0.15)	0.33	-0.68 (1.32)	-0.77 (1.36)	-0.61 (1.29)	0.03 (-0.28, 0.33)	0.86	0.42
3 months	-0.60 (1.37)	-0.58 (1.33)	-0.62 (1.41)	0.07 (-0.23, 0.38)	0.62	-0.71 (1.23)	-0.81 (1.32)	-0.62 (1.14)	-0.14 (-0.45, 0.17)	0.38	0.33
6 months	-0.72 (1.32)	-0.68 (1.39)	-0.75 (1.25)	0.13 (-0.16, 0.41)	0.39	-0.68 (1.19)	-0.77 (1.18)	-0.58 (1.20)	-0.01 (-0.30, 0.28)	0.93	0.51
9 months	-0.86 (1.25)	-0.91 (1.26)	-0.80 (1.25)	-0.02 (-0.30, 0.26)	0.90	-0.89 (1.25)	-1.03 (1.24)	-0.74 (1.26)	-0.17 (-0.45, 0.11)	0.24	0.46
12 months	-0.85 (1.19)	-0.83 (1.20)	-0.88 (1.19)	0.08 (-0.18, 0.34)	0.55	-0.86 (1.21)	-0.92 (1.150	-0.80 (1.27)	-0.10 (-0.37, 0.16)	0.45	0.34
Placental malaria											
	Positive					Negative					
Birth	-1.10 (1.11)	-1.10 (0.98)	-1.10 (1.23)	-0.02 (-0.33, 0.29)	0.37	-0.85 (1.20)	-0.87 (1.22)	-0.82 (1.17)	-0.05 (-0.29, 0.18)	0.67	0.19
1 month	-0.78 (1.28)	-0.81 (1.30)	-0.75 (1.28)	0.07 (-0.30, 0.45)	0.62	-0.62 (1.26)	-0.74 (1.30)	-0.50 (1.21)	-0.10 (-0.40, 0.18)	0.46	0.33
3 months	-0.64 (1.23)	-0.73 (1.24)	-0.56 (1.22)	-0.10 (-0.49, 0.28)	0.60	-0.62 (1.40)	-0.63 (1.40)	-0.61 (1.39)	0.02 (-0.28, 0.32)	0.91	0.39
6 months	-0.62 (1.24)	-0.59 (1.23)	-0.66 (1.25)	0.07 (-0.29, 0.45)	0.91	-0.71 (1.31)	-0.78 (1.33)	-0.62 (1.28)	-0.02 (-0.30, 0.26)	0.91	0.83
9 months	-0.74 (1.25)	-0.78 (1.31)	-0.71 (1.19)	0.04 (-0.32, 0.39)	0.89	-0.94 (1.28)	-1.02 (1.22)	-0.84 (1.33)	-0.09 (-0.36, 0.19)	0.54	0.59
12 months	-0.69 (1.11)	-0.68 (1.06)	-0.69 (1.69)	-0.34 (-0.75, 0.08)	0.96	-0.92 (1.24)	-0.95 (1.22)	-0.88 (1.27)	-0.04 (-0.29, 0.21)	0.77	0.82
Maternal HIV status											

© 2025 Mzembe G et al. *JAMA Network Open*.

eTable 3 (cont): Treatment effects on Growth and Maternal and Infant Characteristics: Subgroup analyses

	Overall Mean (SD) or n/N (%)	FCM Mean (SD) or n/N (%)	SOC Mean (SD)/ n/N (%)	Mean Difference[a] or Risk Ratio[b] (95% CI)	Two-sided P-value	Overall Mean (SD) or n/N (%)	FCM Mean (SD) or n/N (%)	SOC Mean (SD) or n/N (%)	Mean Difference[a] or Risk Ratio[b] (95% CI)	Two-sided P-value	Inter-action P-value
	Positive					Negative					
Birth	-1.82 (1.31)	-0.81 (1.41)	-0.83 (1.20)	0.06 (-0.35, 0.47)	0.76	-0.94 (1.11)	-0.97 (1.08)	-0.92 (1.15)	-0.06 (-0.25, 0.12)	0.50	0.58
1 month	-0.53 (1.21)	-0.75 (1.30)	-0.27 (1.04)	-0.39 (-0.91, 0.14)	0.15	-0.73 (1.28)	-0.79 (1.29)	-0.68 (1.27)	-0.01 (-0.24, 0.22)	0.92	0.20
3 months	-0.75 (1.28)	-0.91 (1.56)	-0.58 (0.89)	-0.37 (-0.90, 0.16)	0.17	-0.62 (1.31)	-0.63 (1.26)	-0.62 (1.35)	0.04 (-0.20, 0.27)	0.77	0.17
6 months	-1.07 (1.38)	-1.24 (1.39)	-0.87 (1.35)	-0.29 (-0.77, 0.20)	0.24	-0.62 (1.22)	-0.59 (1.23)	-0.64 (1.22)	0.15 (-0.07, 0.37)	0.18	0.11
9 months	-1.27 (1.26)	-1.47 (1.27)	-1.03 (1.23)	-0.39 (-0.85, 0.07)	0.10	-0.79 (1.23)	-0.85 (1.21)	-0.73 (1.26)	-0.02 (-0.23, 0.20)	0.88	0.15
12 months	-1.11 (1.15)	-1.24 (1.13)	-0.96 (1.17)	-0.28 (-0.72, 0.16)	0.22	-0.80 (1.21)	-0.80 (1.17)	-0.80 (1.26)	0.04 (-0.16, 0.25)	0.68	0.19
Maternal age											
	Age <20 years					Age ≥20 years					
Birth	-1.14 (1.10)	-1.16 (1.07)	-1.12 (1.13)	-0.06 (-0.30, 0.19)	0.64	-0.74 (1.15)	-0.76 (1.16)	-0.72 (1.14)	-0.03 (-0.26, 0.19)	0.78	0.88
1 month	-0.91 (1.31)	-0.92 (1.40)	-0.91 (1.23)	0.01 (-0.29, 0.32)	0.93	-0.50 (1.19)	-0.66 (1.17)	-0.32 (1.19)	-0.16 (-0.44, 0.12)	0.26	0.40
3 months	-0.76 (1.35)	-0.81 (1.43)	-0.71 (1.28)	-0.06 (-0.37, 0.25)	0.71	-0.52 (1.24)	-0.56 (1.20)	-0.50 (1.28)	-0.03 (-0.32, 0.26)	0.85	0.89
6 months	-0.75 (1.31)	-0.76 (1.38)	-0.74 (1.25)	0.05 (-0.25, 0.36)	0.72	-0.64 (1.23)	-0.68 (1.21	-0.59 (1.25)	0.04 (-0.23, 0.32)	0.77	0.95
9 months	-0.90 (1.30)	-1.00 (1.34)	-0.82 (1.26)	-0.04 (-0.33, 0.24)	0.76	-0.82 (1.21)	-0.93 (1.15)	-0.71 (1.27)	-0.17 (-0.43, 0.10)	0.22	0.54
12 months	-0.90 (1.21)	-0.94 (1.15)	-0.87 (1.27)	-0.03 (-0.30, 0.24)	0.81	-0.80 (1.20)	-0.82 (1.20)	-0.78 (1.21)	-0.03 (-0.27, 0.22)	0.84	0.97
Maternal height											
	Height <150 cm					Height ≥150 cm					
Birth	-1.24 (1.30)	-1.13 (1.35)	-1.33 (1.27)	0.15 (-0.25, 0.56)	0.47	-0.85 (1.10)	-0.90 (1.09)	-0.81 (1.11)	-0.09 (-0.28, 0.91)	0.32	0.28
1 month	-1.12 (1.37)	-1.34 (1.57)	-0.90 (1.10)	-0.28 (-0.77, 0.22)	0.27	-0.60 (1.22)	-0.65 (1.18)	-0.55 (1.26)	-0.03 (-0.25, 0.20)	0.82	0.37
3 months	-1.03 (1.33)	-1.26 (1.36)	-0.81 (1.27)	-0.25 (-0.75, 0.26)	0.34	-0.56 (1.28)	-0.55 (1.28)	-0.56 (1.29)	0.00 (-0.24, 0.23)	1.00	0.39
6 months	-0.99 (1.17)	-1.21 (1.30)	-0.76 (0.99)	-0.27 (-0.76, 0.21)	0.27	-0.62 (1.28)	-0.61 (1.26)	-0.63 (1.30)	0.11 (-0.11, 0.33)	0.33	0.16
9 months	-1.10 (1.07)	-1.26 (1.09)	-0.93 (1.05)	-0.25 (-0.72, 0.21)	0.29	-0.81 (1.29)	-0.90 (1.26)	-0.72 (1.31)	-0.08 (-0.30, 0.13)	0.45	0.51
12 months	-0.95 (1.04)	-1.10 (1.00)	-0.78 (1.08)	-0.24 (-0.68, 0.20)	0.29	-0.83 (1.24)	-0.83 (1.20)	-0.83 (1.27)	0.01 (-0.19, 0.21)	0.92	0.31
Maternal education											
	None/Primary					Secondary/ Tertiary					
Birth	-1.02 (1.18)	-1.08 (1.16)	-0.97 (1.19)	-0.12 (-0.33, 0.10)	0.28	-0.76 (1.09)	-0.69 (1.08)	-0.82 (1.10)	0.15 (-0.14, 0.43)	0.31	0.15
1 month	-0.78 (1.30)	-0.82 (1.36)	-0.74 (1.24)	0.02 (-0.24, 0.29)	0.87	-0.59 (1.19)	-0.74 (1.16)	-0.45 (1.20)	-0.20 (-0.56, 0.16)	0.27	0.32
3 months	-0.70 (1.36)	-0.70 (1.37)	-0.69 (1.35)	0.03 (-0.24, 0.30)	0.83	-0.53 (1.16)	-0.57 (1.14)	-0.51 (1.19)	-0.04 (-0.41, 0.33)	0.85	0.78
6 months	-0.70 (1.29)	-0.75 (1.30)	-0.64 (1.29)	-0.01 (-0.27, 0.24)	0.92	-0.63 (1.20)	-0.64 (1.23)	-0.63 (1.18)	0.15 (-0.19, 0.49)	0.39	0.45
9 months	-0.91 (1.25)	-0.98 (1.23)	-0.84 (1.26)	-0.04 (-0.29, 0.20)	0.74	-0.80 (1.25)	-0.96 (1.24)	-0.64 (1.24)	-0.24 (-0.57, 0.08)	0.15	0.33
12 months	-0.86 (1.16)	-0.86 (1.12)	-0.87 (1.20)	0.02 (-0.21, 0.25)	0.84	-0.85 (1.27)	-0.91 (1.27)	-0.79 (1.26)	-0.05 (-0.36, 0.25)	0.72	0.69
Infant sex											
	Female					Male					
Birth	-0.88 (1.08)	-0.90 (1.09)	-0.85 (1.07)	-0.07 (-0.31, 0.18)	0.60	-0.96 (1.20)	-0.97 (1.18)	-0.94 (1.23)	-0.01 (-0.25, 0.22)	0.91	0.76
1 month	-0.60 (1.21)	-0.62 (1.12)	-0.57 (1.30)	0.02 (-0.28, 0.31)	0.92	-0.79 (1.31)	-0.93 (1.42)	-0.66 (1.18)	-0.13 (-0.43, 0.16)	0.37	0.49
3 months	-0.52 (1.20)	-0.51 (1.12)	-0.54 (1.26)	-0.03 (-0.33, 0.28)	0.86	-0.75 (1.39)	-0.83 (1.46)	-0.67 (1.31)	-0.04 (-0.34, 0.26)	0.80	0.96
6 months	-0.57 (1.21)	-0.56 (1.20)	-0.57 (1.23)	0.10 (-0.19, 0.39)	0.49	-0.81 (1.32)	-0.87 (1.36)	-0.74 (1.27)	0.01 (-0.28, 0.29)	0.96	0.64
9 months	-0.70 (1.18)	-0.77 (1.11)	-0.62 (1.25)	-0.08 (-0.36, 0.20)	0.56	-1.03 (1.30)	-1.17 (1.33)	-0.89 (1.27)	-0.12 (-0.39, 0.16)	0.40	0.86
12 months	-0.66 (1.12)	-0.66 (1.04)	-0.67 (1.19)	0.00 (-0.26, 0.26)	0.99	-1.03 (1.26)	-1.08 (1.25)	-0.97 (1.27)	-0.04 (-0.29, 0.22)	0.77	0.83
**MEAN WEIGHT FOR LENGTH (WLZ)**											
Iron status at randomization											
	Iron deficient					Non-deficient					
Birth	-0.50 (1.72)	-0.49 (1.70)	-0.52 (1.75)	-0.02 (-0.46, 0.42)	0.93	-0.75 (1.88)	-0.85 (1.99)	-0.66 (1.77)	-0.20 (-0.59, 0.19)	0.31	0.54
1 month	0.14 (2.17)	-0.17 (2.18)	0.46 (2.12)	-0.57 (-1.13, -0.02)	0.04	0.22 (1.94)	0.16 (1.82)	0.27 (2.04)	-0.07 (-0.54, 0.40)	0.76	0.18
3 months	0.25 (1.99)	-0.07 (1.97)	0.55 (1.97)	-0.62 (-1.14, -0.10)	0.02	0.40 (1.86)	0.44 (1.84)	0.36 (1.90)	0.07 (-0.35, 0.50)	0.73	0.04
6 months	0.02 (1.73)	-0.18 (1.75)	0.22 (1.70)	-0.33 (-0.76, 0.10)	0.13	0.21 (1.68)	0.26 (1.79)	0.16 (1.57)	0.17 (-0.19, 0.54)	0.35	0.08

© 2025 Mzembe G et al. *JAMA Network Open*.

eTable 3 (cont): Treatment effects on Growth and Maternal and Infant Characteristics: Subgroup analyses

	Overall Mean (SD) or n/N (%)	FCM Mean (SD) or n/N (%)	SOC Mean (SD)/ n/N (%)	Mean Difference[a] or Risk Ratio[b] (95% CI)	Two-sided P-value	Overall Mean (SD) or n/N (%)	FCM Mean (SD) or n/N (%)	SOC Mean (SD) or n/N (%)	Mean Difference[a] or Risk Ratio[b] (95% CI)	Two-sided P-value	Inter-action P-value
9 months	-0.31 (1.52)	-0.52 (1.42)	-0.08 (1.60)	-0.46 (-0.86, -0.06)	0.02	-0.09 (1.68)	-0.14 (1.62)	-0.04 (1.75)	-0.04 (-0.38, 0.30)	0.82	0.11
12 months	-0.33 (1.55)	-0.29 (1.46)	-0.38 (1.67)	0.12 (-0.23, 0.47)	0.50	-0.23 (1.42)	-0.23 (1.44)	-0.24 (1.43)	-0.03 (-0.33, 0.27)	0.86	0.53
Inflammation at randomization											
	Yes					No					
Birth	-0.76 (1.89)	-0.87 (2.04)	-0.65 (1.74)	-0.27 (-0.68, 0.13)	0.19	-0.52 (1.72)	-0.50 (1.68)	-0.53 (1.78)	0.03 (-0.39, 0.45)	0.901	0.31
1 month	0.12 (2.09)	-0.05 (2.07)	0.28 (2.09)	-0.32 (-0.81, 0.18)	0.21	0.26 (1.98)	0.10 (1.89)	0.41 (2.06)	-0.23 (-0.75, 0.28)	0.38	0.82
3 months	0.37 (2.02)	0.25 (1.86)	0.49 (2.16)	-0.34 (-0.81, 0.12)	0.15	0.30 (1.80)	0.21 (1.95)	0.37 (1.66)	-0.07 (-0.54, 0.41)	0.78	0.41
6 months	0.08 (1.75)	0.03 (1.80)	0.14 (1.69)	-0.08 (-0.47, 0.30)	0.67	0.18 (1.66)	0.14 (1.77)	0.23 (1.55)	0.01 (-0.38, 0.42)	0.93	0.71
9 months	-0.12 (1.46)	-0.19 (1.53)	-0.04 (1.39)	-0.18 (-0.54, 0.18)	0.33	-0.24 (1.76)	-0.41 (1.56)	-0.07 (1.94)	-0.26 (-0.63, 0.11)	0.17	0.78
12 months	-0.22 (1.43)	0.16 (1.38)	-0.29 (1.49)	0.09 (-0.22, 0.42)	0.57	-0.32 (1.53)	-0.34 (1.51)	-0.31 (1.55)	-0.02 (-0.34, 0.30)	0.89	0.62
Placental malaria											
	Positive					Negative					
Birth	-0.69 (1.90)	-0.75 (1.91)	-0.62 (1.90)	-0.18 (-0.70, 0.35)	0.51	-0.58 (1.81)	-0.56 (1.71)	-0.57 (1.77)	-0.09 (-0.43, 0.26)	0.62	0.78
1 month	0.22 (1.81)	0.13 (1.67)	0.30 (1.94)	-0.19 (-0.82, 0.45)	0.57	0.19 (2.15)	0.26 (2.05)	0.44 (2.14)	-0.34 (-0.76, 0.08)	0.12	0.70
3 months	0.32 (1.77)	0.34 (1.95)	0.30 (1.58)	-0.06 (-0.63, 0.51)	0.83	0.43 (2.05)	0.44 (1.94)	0.69 (2.17)	-0.28 (-0.67, 0.11)	0.16	0.54
6 months	0.24 (1.77)	0.35 (2.01)	0.14 (1.50)	0.01 (-0.47, 0.49)	1.00	0.12 (1.70)	0.21 (1.68)	0.33 (1.71)	-0.09 (-0.43, 0.24)	0.58	0.73
9 months	-0.11 (1.48)	-0.02 (1.52)	-0.21 (1.46)	-0.11 (-0.56, 0.34)	0.63	-0.21 (1.73)	-0.08 (1.82)	-0.01 (1.95)	-0.28 (-0.59, 0.03)	0.075	0.53
12 months	-0.09 (1.31)	0.06 (1.15)	-0.23 (1.45)	0.14 (-0.26, 0.53)	0.50	-0.31 (1.53)	-0.28 (1.56)	-0.28 (1.55)	-0.03 (-0.30, 0.24)	0.81	0.49
Maternal HIV status											
	Positive					Negative					
Birth	-058 (1.85)	-0.58 (1.88)	-0.57 (1.81)	0.00 (-0.68, 0.68)	1.00	-0.64 (1.81)	-0.68 (1.87)	-0.60 (1.74)	-0.12 (-0.44, 0.20)	0.47	0.76
1 month	-0.02 (1.99)	-0.32 (2.14)	0.28 (1.83)	-0.44 (-1.33, 0.46)	0.34	0.24 (2.03)	0.10 (1.95)	0.37 (2.11)	-0.25 (-0.64, 0.14)	0.21	0.70
3 months	-0.01 (1.37)	-0.19 (1.55)	0.18 (1.19)	-0.42 (-1.22, 0.38)	0.30	0.42 (1.98)	0.34 (1.92)	0.49 (2.03)	-0.15 (-0.51, 0.20)	0.40	0.55
6 months	-0.29 (1.75)	-0.56 (1.67)	-0.02 (1.83)	-0.35 (-1.00, 0.31)	0.30	0.24 (1.68)	0.24 (1.78)	0.24 (1.58)	0.02 (-0.28, 0.32)	0.88	0.32
9 months	-0.62 (1.48)	-0.84 (1.50)	-0.42 (1.45)	-0.39 (-0.99, 0.22)	0.21	-0.08 (1.62)	-0.17 (1.51)	0.02 (1.72)	-0.18 (-0.46, 0.11)	0.22	0.53
12 months	-0.53 (1.39)	-0.74 (1.27)	-0.31 (1.51)	-0.40 (-0.93, 0.13)	0.14	-0.21 (1.49)	-0.14 (1.44)	-0.27 (1.54)	0.11 (-0.14, 0.36)	0.38	0.09
Maternal age											
	Age <20 years					Age ≥20 years					
Birth	-0.78 (1.85)	-0.79 (1.94)	-0.76 (1.76)	-0.09 (-0.53, 0.34)	0.68	-0.51 (1.77)	-0.57 (1.79)	-0.45 (1.75)	-0.14 (-0.52, 0.24)	0.47	0.87
1 month	0.22 (1.73)	0.32 (1.74)	0.12 (1.76)	0.22 (-0.29, 0.73)	0.41	0.42 (2.22)	-0.20 (2.13)	0.63 (2.31)	-0.77 (-1.25, -0.29)	0.001	0.01
3 months	0.53 (1.82)	0.42 (1.97)	0.64 (1.66)	-0.25 (-0.71, 0.22)	0.30	0.16 (1.25)	0.08 (1.77)	0.24 (2.12)	-0.16 (-0.61, 0.28)	0.47	0.80
6 months	0.31 (1.75)	0.24 (1.91)	0.37 (1.58)	-0.12 (-0.53, 0.29)	0.57	0.02 (1.66)	-0.04 (1.67)	0.07 (1.65)	-0.01 (-0.38, 0.37)	0.98	0.69
9 months	-0.07 (1.69)	-0.21 (1.61)	0.07 (1.76)	-0.24 (-0.61, 0.14)	0.22	-0.25 (1.54)	-0.36 (1.47)	-0.14 (1.60)	-0.22 (-0.57, 0.13)	0.21	0.96
12 months	-0.20 (1.49)	-0.19 (1.46)	-0.21 (1.51)	0.02 (-0.31, 0.35)	0.90	0.02 (1.48)	-0.28 (1.41)	0.32 (1.54)	0.02 (-0.28, 0.32)	0.90	1.00
Maternal height											
	Height <150 cm					Height ≥150 cm					
Birth	-0.68 (1.83)	-0.61 (1.78)	-0.75 (1.87)	0.00 (-0.72, 0.73)	0.99	-0.62 (1.81)	-0.67 (1.88)	-0.56 (1.74)	-0.14 (-0.45, 0.18)	0.40	0.73
1 month	0.10 (1.96)	-0.19 (2.44)	0.38 (1.48)	-0.54 (-1.37, 0.30)	0.21	0.23 (2.03)	0.08 (1.87)	0.37 (2.18)	-0.25 (-0.64, 0.14)	0.20	0.54
3 months	0.44 (1.90)	0.22 (1.82)	0.66 (1.98)	-0.57 (-1.34, 0.20)	0.15	0.32 (1.89)	0.24 (1.88)	0.39 (1.90)	-0.13 (-0.49, 0.22)	0.47	0.31
6 months	0.23 (1.73)	-0.13 (1.81)	0.59 (1.64)	-0.71 (-1.37, -0.06)	0.03	0.13 (1.76)	0.13 (1.78)	0.13 (1.61)	0.07 (-0.23, 0.38)	0.63	0.03
9 months	-0.20 (1.35)	-0.41 (1.33)	0.02 (1.36)	-0.59 (-1.20, 0.01)	0.06	-0.16 (1.66)	-0.27 (1.57)	-0.05 (1.75)	-0.15 (-0.43, 0.13)	0.28	0.20
12 months	-0.14 (1.45)	-0.25 (1.31)	-0.03 (1.58)	-0.36 (-0.90, 0.17)	0.19	-0.28 (1.49)	-0.24 (1.46)	-0.32 (1.51)	0.10 (-0.15, 0.34)	0.44	0.13
Maternal education											
	None/Primary					Secondary/ Tertiary					
Birth	-0.37 (1.92)	-0.67 (1.98)	-0.64 (1.85)	-0.07 (-0.45, 0.31)	0.71	-0.61 (1.68)	-0.66 (1.70)	-0.55 (1.65)	-0.10 (-0.58, 0.37)	0.67	0.92
1 month	0.25 (2.00)	0.08 (1.98)	0.41 (2.02)	-0.29 (-0.73, 0.16)	0.21	0.14 (2.11)	-0.04 (2.07)	0.32 (2.15)	-0.26 (-0.88, 0.36)	0.41	0.94

© 2025 Mzembe G et al. *JAMA Network Open*.

eTable 3 (cont): Treatment effects on Growth and Maternal and Infant Characteristics: Subgroup analyses

	Overall Mean (SD) or n/N (%)	FCM Mean (SD) or n/N (%)	SOC Mean (SD)/ n/N (%)	Mean Difference[a] or Risk Ratio[b] (95% CI)	Two-sided P-value	Overall Mean (SD) or n/N (%)	FCM Mean (SD) or n/N (%)	SOC Mean (SD) or n/N (%)	Mean Difference[a] or Risk Ratio[b] (95% CI)	Two-sided P-value	Inter-action P-value
3 months	0.41 (1.96)	0.36 (1.93)	0.45 (1.99)	-0.09 (-0.49, 0.31)	0.66	0.16 (1.73)	0.01 (1.72)	0.29 (1.73)	-0.27 (-0.83, 0.30)	0.36	0.62
6 months	0.24 (1.60)	0.17 (1.60)	0.31 (1.59)	-0.11 (-0.46, 0.24)	0.54	-0.01 (1.05)	-0.03 (2.07)	0.02 (1.64)	0.08 (-0.39, 0.55)	0.74	0.53
9 months	-0.10 (1.61)	-0.21 (1.46)	0.01 (1.76)	-0.18 (-0.50, 0.14)	0.28	-0.32 (1.62)	-0.48 (1.65)	-0.15 (1.59)	-0.32 (-0.75, 0.12)	0.16	0.62
12 months	-0.19 (1.41)	-0.12 (1.37)	-0.25 (1.44)	0.09 (-0.19, 0.37)	0.53	-0.40 (1.57)	-0.44 (1.50)	-0.36 (1.64)	-0.05 (-0.42, 0.33)	0.80	0.57
Infant sex											
	Female					Male					
Birth	-0.71 (1.73)	-0.80 (1.85)	-0.61 (1.60)	-0.24 (-0.66, 0.17)	0.25	-0.55 (1.89)	-0.54 (1.87)	-0.56 (1.90)	0.00 (-0.40, 0.40)	0.99	0.41
1 month	0.19 (1.94)	0.15 (2.03)	0.22 (1.84)	-0.04 (-0.54, 0.46)	0.87	0.22 (2.11)	-0.09 (1.93)	0.53 (2.27)	-0.56 (-1.05, 0.06)	0.03	0.15
3 months	0.38 (1.89)	0.37 (1.91)	0.38 (1.86)	-0.06 (-0.52, 0.40)	0.81	0.31 (1.90)	0.12 (1.83)	0.50 (1.97)	-0.35 (-0.80, 0.10)	0.13	0.37
6 months	0.21 (1.75)	0.28 (1.85)	0.13 (1.64)	0.18 (-0.21, 0.57)	0.36	0.10 (1.66)	-0.10 (1.70)	0.29 (1.61)	-0.30 (-0.68, 0.09)	0.13	0.09
9 months	-0.11 (1.59)	-0.24 (1.40)	0.02 (1.78)	-0.19 (-0.56, 0.17)	0.30	-0.23 (1.62)	-0.35 (1.65)	-0.10 (1.59)	-0.26 (-0.61, 0.10)	1.16	0.81
12 months	-0.17 (1.38)	-0.07 (1.32)	-0.27 (1.43)	0.20 (-0.12, 0.52)	0.22	-0.34 (1.57)	-0.40 (1.51)	-0.27 (1.62)	-0.15 (-0.46, 0.16)	0.35	0.13
**STUNTING**											
Iron status at randomization											
	Iron deficient					Non-deficient					
Birth	64/295 (21.7)	42/155 (27.1)	22/140 (15.7)	1.73 (1.09, 2.76)	0.02	108/395 (27.3)	49/191 (25.7)	59/204 (28.9)	0.91 (0.65, 1.26)	0.57	0.03
1 month	42/202 (20.8)	20/103 (19.4)	22/99 (22.2)	0.87 (0.51, 1.49)	0.62	82/294 (27.9)	41/139 (29.5)	41/155 (26.5)	1.10 (0.76, 1.59)	0.60	0.48
3 months	35/196 (17.9)	13/97 (13.4)	22/99 (22.2)	0.61 (0.33, 1.14)	0.12	78/290 (26.9)	36/140 (25.7)	42/150 (28.0)	0.90 (0.61, 1.31)	0.57	0.31
6 months	43/225 (19.1)	21/112 (18.8)	22/113 (19.5)	0.96 (0.56, 1.64)	0.87	81/322 (25.2)	39/163 (23.9)	42/159 (26.4)	0.88 (0.60, 1.28)	0.49	0.79
9 months	89/306 (29.1)	27/115 (23.5)	30/107 (28.0)	0.83 (0.53, 1.30)	0.42	57/222 (25.7)	47/154 (30.5)	42/152 (27.6)	1.07 (0.75, 1.52)	0.72	0.39
12 months	64/236 (27.1)	34/123 (27.6)	30/113 (26.6)	1.05 (0.69, 1.60)	0.82	93/319 (29.2)	51/163 (31.3)	42/156 (26.9)	1.14 (0.80, 1.61)	0.47	0.78
Inflammation at randomization											
	Yes					No					
Birth	92/357 (25.8)	54/180 (30.0)	38/177 (21.5)	1.42 (0.98, 2.05)	0.06	80/333 (24.02)	37/166 (22.3)	43/167 (25.8)	0.88 (0.60, 1.29)	0.51	0.08
1 month	61/252 (24.2)	35/126 (27.8)	26/126 (20.6)	1.36 (0.88, 2.11)	0.17	63/244 (25.8)	26/116 (22.4)	37/128 (28.9)	0.76 (0.49, 1.17)	0.21	0.06
3 months	53/247 (21.5)	19/121 (15.7)	34/126 (27.0)	0.57 (0.34, 0.95)	0.03	60/239 (25.1)	30/116 (25.9)	30/123 (24.4)	1.05 (0.68, 1.63)	0.82	0.07
6 months	60/279 (21.5)	30/142 (21.1)	30/137 (21.9)	0.95 (0.61, 1.49)	0.83	64/268 (23.9)	30/133 (22.6)	34/135 (25.2)	0.86 (0.56, 1.32)	0.50	0.75
9 months	80/267 (30.0)	35/137 (25.6)	45/130 (34.6)	0.71 (0.49, 1.04)	0.08	66/261 (25.3)	39/132 (29.6)	27/129 (20.9)	1.38 (0.90, 2.11)	0.14	0.02
12 months	83/275 (30.2)	43/143 (30.1)	40/132 (30.3)	0.98 (0.68, 1.41)	0.91	74/280 (26.4)	42/143 (29.4)	32/137 (23.4)	1.24 (0.83, 1.85)	0.29	0.39
Placental malaria											
	Positive					Negative					
Birth	54/214 (25.2)	27/105 (25.7)	27/109 (24.8)	0.97 (0.61, 1.56)	0.91	123/492 (25.0)	57/244 (23.4)	66/248 (26.6)	1.16 (0.85, 1.58)	0.35	0.56
1 month	48/157 (30.6)	23/81 (28.4)	25/76 (32.9)	1.10 (0.69, 1.77)	0.68	81/354 (22.9)	42/178 (23.6)	39/176 (22.2)	0.96 (0.65, 1.40)	0.82	0.64
3 months	38/159 (23.9)	21/82 (25.6)	17/77 (22.1)	0.81 (0.46, 1.42)	0.46	77/342 (22.5)	44/172 (25.6)	33/170 (19.4)	0.77 (0.52, 1.15)	0.20	0.89
6 months	44/183 (24.0)	22/90 (24.4)	22/93 (23.7)	0.93 (0.56, 1.57)	0.80	84/380 (22.1)	44/188 (23.4)	40/192 (20.8)	0.88 (0.60, 1.28)	0.49	0.84
9 months	50/180 (27.8)	25/87 (28.7)	25/93 (26.9)	0.89 (0.56, 1.44)	0.65	101/363 (27.8)	48/177 (27.1)	53/186 (28.5)	1.03 (0.74, 1.44)	0.85	0.64
12 months	52/184 (28.3)	20/86 (23.3)	32/98 (33.0)	1.34 (0.83, 2.18)	0.23	111/386 (28.8)	53/188 (28.2)	58/198 (29.3)	1.04 (0.76, 1.43)	0.79	0.39
Maternal HIV status											
	Positive					Negative					
Birth	25/119 (21.0)	16/59 (27.1)	09/60 (15.0)	1.83 (0.88, 3.83)	0.11	150/581 (25.8)	77/296 (26.0)	73/285 (25.6)	1.03 (0.78, 1.36)	0.83	0.15
1 month	17/80 (21.3)	07/44 (15.9)	10/36 (27.8)	0.59 (0.25, 1.40)	0.23	111/426 (26.1)	57/206 (27.7)	54/220 (24.6)	1.11 (0.81, 1.53)	0.51	0.18
3 months	17/81 (20.1)	08/42 (19.1)	09/39 (23.1)	0.87 (0.38, 2.02)	0.75	98/416 (23.6)	42/204 (20.6)	56/212 (26.4)	0.76 (0.54, 1.08)	0.13	0.78
6 months	20/98 (20.4)	11/54 (20.4)	09/44 (20.5)	1.03 (0.47, 2.25)	0.95	108/461 (23.4)	51/230 (22.2)	57/231 (24.7)	0.87 (0.62, 1.21)	0.41	0.70
9 months	30/97 (30.9)	19/53 (35.9)	11/44 (25.0)	1.47 (0.78, 2.76)	0.23	121/442 (27.4)	59/225 (26.2)	62/217 (28.6)	0.86 (0.65, 1.20)	0.43	0.15
12 months	35/100 (35.0)	17/52 (32.7)	18/48 (37.5)	0.91 (0.53, 1.57)	0.75	127/466 (27.3)	72/243 (29.6)	55/223 (24.7)	1.17 (0.86, 1.59)	0.30	0.43
Maternal age											

© 2025 Mzembe G et al. *JAMA Network Open*.

eTable 3 (cont): Treatment effects on Growth and Maternal and Infant Characteristics: Subgroup analyses

	Overall Mean (SD) or n/N (%)	FCM Mean (SD) or n/N (%)	SOC Mean (SD)/ n/N (%)	Mean Difference[a] or Risk Ratio[b] (95% CI)	Two-sided P-value	Overall Mean (SD) or n/N (%)	FCM Mean (SD) or n/N (%)	SOC Mean (SD) or n/N (%)	Mean Difference[a] or Risk Ratio[b] (95% CI)	Two-sided P-value	Inter-action P-value
	Age <20 years					Age ≥20 years					
Birth	87/315 (27.6)	44/156 (28.2)	43/159 (27.0)	1.07 (0.75, 1.54)	0.70	90/391 (23.0)	49/201 (24.4)	41/190 (21.6)	1.14 (0.79, 1.64)	0.50	0.83
1 month	73/245 (29.8)	40/114 (35.1)	33/131 (25.2)	1.40 (0.95, 2.05)	0.09	56/266 (21.1)	24/138 (17.4)	32/128 (25.0)	0.69 (0.43, 1.10)	0.12	0.02
3 months	72/244 (29.5)	32/118 (27.1)	40/126 (31.8)	0.83 (0.57, 1.24)	0.37	43/257 (16.7)	18/129 (14.0)	25/128 (19.5)	0.73 (0.42, 1.26)	0.26	0.68
6 months	79/257 (30.7)	39/128 (30.5)	40/129 (31.0)	0.95 (0.65, 1.37)	0.76	49/306 (16.0)	23/157 (14.7)	26/149 (17.5)	0.84 (0.50, 1.40)	0.50	0.71
9 months	80/256 (31.3)	39/128 (30.5)	41/128 (32.0)	0.90 (0.62, 1.29)	0.56	71/287 (24.7)	39/151 (25.8)	32/136 (23.5)	1.12 (0.74, 1.67)	0.59	0.43
12 months	85/263 (32.3)	46/136 (33.8)	39/127 (30.7)	1.06 (0.74, 1.51)	0.76	78/307 (25.4)	44/160 (27.5)	34/147 (23.1)	1.22 (0.83, 1.80)	0.31	0.59
Maternal height											
	Height <150 cm					Height ≥150 cm					
Birth	40/121 (33.1)	16/57 (28.1)	24/64 (37.5)	0.77 (0.45, 1.31)	0.34	137/585 (23.4)	77/300 (25.7)	60/285 (21.1)	1.24 (0.92, 1.67)	0.17	0.13
1 month	28/92 (30.4)	15/46 (32.6)	13/46 (28.3)	1.34 (0.61, 2.12)	0.68	101/419 (24.1)	49/206 (23.8)	52/213 (24.4)	0.98 (0.70, 1.37)	0.89	0.68
3 months	31/88 (35.2)	15/43 (34.9)	16/45 (35.6)	0.94 (0.53, 1.66)	0.84	84/413 (20.3)	35/204 (17.2)	49/209 (23.4)	0.73 (0.50, 1.08)	0.12	0.48
6 months	32/98 (32.7)	17/50 (34.0)	15/48 (31.3)	1.04 (0.59, 1.85)	0.88	96/465 (20.7)	45/235 (19.2)	51/230 (22.2)	0.85 (0.60, 1.21)	0.37	0.55
9 months	34/98 (34.7)	14/49 (28.6)	20/49 (40.8)	0.67 (0.38, 1.19)	0.17	117/445 (26.3)	64/230 (27.8)	53/215 (24.7)	1.11 (0.81, 1.51)	0.53	0.13
12 months	36/99 (36.4)	23/52 (44.2)	13/47 (27.7)	1.58 (0.90, 2.74)	0.11	127/471 (26.9)	67/244 (27.5)	60/227 (26.4)	1.03 (0.76, 1.39)	0.84	0.19
Maternal education											
	None/Primary					Secondary/ Tertiary					
Birth	124/430 (28.8)	63/219 (28.8)	61/211 (28.9)	1.02 (0.75, 1.37)	0.92	47/251 (18.7)	26/126 (20.6)	21/125 (16.8)	1.23 (0.73, 2.08)	0.44	0.53
1 month	83/325 (25.5)	42/166 (25.3)	41/159 (25.8)	0.97 (0.67, 1.40)	0.86	44/167 (26.4)	22/78 (28.2)	22/89 (24.7)	1.15 (0.69, 1.91)	0.59	0.59
3 months	84/323 (26.0)	35/164 (21.3)	49/159 (30.8)	0.69 (0.47, 1.01)	0.05	24/161 (14.9)	12/76 (15.8)	12/85 (14.1)	1.09 (0.52, 2.29)	0.81	0.28
6 months	91/350 (26.0)	47/183 (25.7)	44/167 (26.4)	0.95 (0.67, 1.36)	0.80	32/192 (16.7)	14/93 (15.1)	18/99 (18.2)	0.80 (0.42, 1.51)	0.49	0.63
9 months	110/342 (32.2)	55/180 (30.6)	55/162 (34.0)	0.88 (0.64, 1.20)	0.41	36/185 (19.5)	20/92 (21.7)	16/93 (17.2)	1.24 (0.69, 2.23)	0.48	0.31
12 months	112/354 (31.6)	63/188 (33.5)	49/166 (29.5)	1.12 (0.82, 1.54)	0.46	46/195 (23.6)	25/99 (25.3)	21/96 (21.9)	1.13 (0.68, 1.89)	0.62	0.98
Infant sex											
	Female					Male					
Birth	103/363 (28.4)	38/171 (22.2)	36/172 (20.9)	1.08 (0.72, 1.62)	0.71	46/195 (23.6)	55/186 (29.6)	48/177 (27.1)	1.10 (0.79, 1.54)	0.56	0.94
1 month	71/255 (27.8)	28/125 (22.4)	30/131 (22.9)	0.98 (0.62, 1.53)	0.92	74/343 (21.6)	36/127 (28.4)	35/128 (27.3)	1.03 (0.69, 1.53)	0.88	0.86
3 months	63/256 (24.6)	22/117 (18.8)	30/128 (23.4)	0.81 (0.50, 1.32)	0.40	58/256 (22.7)	28/130 (21.5)	35/126 (27.8)	0.76 (0.50, 1.17)	0.21	0.85
6 months	78/281 (27.8)	24/141 (17.0)	26/141 (18.4)	0.91 (0.55, 1.50)	0.71	52/245 (21.2)	38/144 (26.4)	40/137 (29.2)	0.88 (0.60, 1.28)	0.50	0.91
9 months	98/274 (35.8)	27/140 (19.3)	26/129 (20.2)	0.94 (0.58, 1.52)	0.81	50/282 (17.7)	51/139 (36.7)	47/135 (34.8)	1.02 (0.74, 1.40)	0.92	0.80
12 months	106/289 (36.7)	31/143 (21.7)	26/138 (18.8)	1.15 (0.72, 1.83)	0.56	53/269 (19.7)	59/153 (38.6)	47/136 (34.6)	1.09 (0.80, 1.49)	0.57	0.86
**UNDERWEIGHT**											
Iron status at randomization											
	Iron deficient					Non-deficient					
Birth	31/300 (10.3)	19/156 (12.2)	12/144 (8.3)	1.48 (0.73, 2.99)	0.28	69/413 (16.7)	37/203 (18.2)	32/210 (15.2)	1.22 (0.77, 1.93)	0.39	0.66
1 month	21/202 (10.4)	12/103 (11.7)	9/99 (9.1)	1.25 (0.54, 2.89)	0.60	45/294 (15.3)	25/139 (18.0)	20/155 (12.9)	1.27 (0.75, 2.16)	0.37	0.97
3 months	19/196 (9.7)	10/97 (10.3)	9/99 (9.1)	1.22 (0.52, 2.88)	0.65	51/290 (17.6)	20/140 (14.3)	31/150 (20.7)	0.64 (0.39, 1.06)	0.09	0.20
6 months	33/225 (14.67)	24/112 (21.4)	9/113 (8.0)	2.66 (1.30, 5.43)	0.01	51/322 (15.8)	24/163 (14.7)	27/159 (17.0)	0.80 (0.45, 1.32)	0.38	0.01
9 months	35/222 (15.8)	21/115 (18.3)	14/107 (13.1)	1.51 (0.81, 2.82)	0.19	52/306 (17.0)	27/154 (17.5)	25/152 (16.5)	0.97 (0.59, 1.57)	0.89	0.28
12 months	40/236 (17.0)	19/123 (15.5)	21/113 (18.6)	0.87 (0.50, 1.51)	0.61	44/319 (13.8)	22/163 (13.5)	22/156 (14.1)	0.93 (0.54, 1.61)	0.81	0.85
Inflammation at randomization											
	Yes					No					
Birth	62/366 (16.9)	36/184 (19.6)	26/182 (14.3)	1.46 (0.90, 2.40)	0.13	38/347 (11.0)	18/172 (10.5)	20/175 (11.4)	1.02 (0.55, 1.90)	0.93	0.37
1 month	36/252 (14.3)	20/126 (15.9)	16/126 (12.7)	1.26 (0.69, 2.32)	0.45	30/244 (12.3)	17/116 (14.7)	13/128 (10.2)	1.24 (0.63, 2.43)	0.53	0.97
3 months	37/247 (16.0)	13/121 (10.7)	24/126 (19.1)	0.58 (0.31, 1.11)	0.10	33/239 (13.8)	17/116 (14.7)	16/123 (13.0)	1.02 (0.54, 1.91)	0.95	0.23
6 months	51/279 (18.3)	28/142 (19.7)	23/137 (16.8)	1.19 (0.72, 1.98)	0.50	33/268 (12.3)	20/133 (15.0)	13/135 (9.6)	1.33 (0.69, 2.55)	0.39	0.80

© 2025 Mzembe G et al. *JAMA Network Open*.

eTable 3 (cont): Treatment effects on Growth and Maternal and Infant Characteristics: Subgroup analyses

	Overall Mean (SD) or n/N (%)	FCM Mean (SD) or n/N (%)	SOC Mean (SD)/ n/N (%)	Mean Difference[a] or Risk Ratio[b] (95% CI)	Two-sided P-value	Overall Mean (SD) or n/N (%)	FCM Mean (SD) or n/N (%)	SOC Mean (SD) or n/N (%)	Mean Difference[a] or Risk Ratio[b] (95% CI)	Two-sided P-value	Inter-action P-value
9 months	47/267 (17.6)	24/137 (17.5)	23/130 (17.7)	1.04 (0.62, 1.75)	0.87	40/261 (15.3)	24/132 (18.2)	16/129 (12.4)	1.27 (0.71, 2.28)	0.41	0.61
12 months	46/275 (16.7)	23/143 (16.1)	23/132 (17.4)	0.98 (0.58, 1.67)	0.95	38/280 (13.6)	18/143 (12.6)	20/137 (14.6)	0.79 (0.45, 1.40)	0.42	0.58
Placental malaria											
			Positive					Negative			
Birth	43/223 (19.3)	24/106 (22.6)	19/117 (16.2)	0.66 (0.37, 1.16)	0.15	59/507 (11.6)	21/254 (8.3)	38/253 (15.0)	1.94 (1.15, 3.26)	0.013	0.006
1 month	23/157 (14.6)	10/81 (12.4)	13/76 (17.1)	1.08 (0.51, 2.30)	0.75	45/354 (12.7)	19/178 (10.7)	26/176 (14.8)	1.45 (0.83, 2.53)	0.19	0.54
3 months	29/159 (18.2)	16/82 (19.5)	13/77 (16.9)	0.70 (0.36, 1.34)	0.28	42/342 (12.3)	24/172 (14.0)	18/170 (10.6)	0.83 (0.47, 1.47)	0.52	0.70
6 months	28/183 (15.3)	12/90 (13.3)	16/93 (17.2)	1.09 (0.54, 2.18)	0.82	59/380 (15. 5)	26/188 (13.8)	33/192 (17.2)	1.25 (0.77, 2.01)	0.37	0.75
9 months	24/180 (13.3)	9/87 (10.3)	15/93 (16.1)	1.22 (0.57, 2.64)	0.61	66/363 (18.2)	31/177 (17.5)	35/186 (18.8)	1.16 (0.75, 1.80)	0.50	0.91
12 months	28/184 (15.2)	14/86 (16.3)	14/98 (14.3)	0.72 (0.36, 1.41)	0.34	59/386 (15.3)	30/188 (16.0)	29/198 (14.7)	1.01 (0.64, 1.60)	0.96	0.41
Maternal HIV status											
			Positive					Negative			
Birth	18/123 (14.6)	12/61 (19.7)	6/62 (9.7)	1.89 (0.73, 4.92)	0.19	83/601 (13.8)	45/307 (14.7)	38/294 (12.9)	1.18 (0.77, 1.79)	0.44	0.37
1 month	8/80 (10.0)	6/44 (13.6)	2/36 (5.6)	2.12 (0.45, 10.05)	0.34	60/426 (14.1)	33/206 (16.0)	27/220 (12.3)	1.27 (0.79, 2.03)	0.32	0.54
3 months	10/81 (12.3)	8/42 (19.1)	2/39 (5.1)	3.67 (0.82, 16.52)	0.09	61/416 (14.7)	23/204 (11.3)	38/212 (17.9)	0.63 (0.39, 1.00)	0.05	0.03
6 months	22/98 (22.4)	14/54 (25.9)	8/44 (18.2)	1.30 (0.59, 2.88)	0.51	65/461 (14.1)	35/230 (15.0)	30/231 (13.0)	1.14 (0.72, 1.79)	0.58	0.77
9 months	26/97 (26.8)	16/53 (30.2)	10/44 (22.7)	1.28 (0.63, 2.60)	0.49	64/442 (14.5)	34/225 (15.1)	30/217 (13.8)	1.07 (0.69, 1.68)	0.75	0.68
12 months	20/100 (20.0)	11/52 (21.2)	9/48 (18.8)	1.12 (0.51, 2.45)	0.78	67/466 (14.4)	32/243 (13.2)	35/223 (15.7)	0.85 (0.55, 1.31)	0.45	0.54
Maternal age											
			Age <20 years					Age ≥20 years			
Birth	54/330 (16.4)	26/166 (15.7)	28/164 (17.1)	0.98 (0.59, 1.62)	0.93	48/400 (12.0)	31/204 (15.2)	17/196 (8.7)	1.72 (0.97, 3.04)	0.06	0.14
1 month	43/245 (17.6)	21/114 (18.4)	22/131 (16.8)	1.13 (0.66, 1.95)	0.65	25/266 (9.4)	18/138 (13.0)	7/128 (5.5)	2.11 (0.92, 4.85)	0.08	0.22
3 months	39/244 (16.0)	16/118 (13.6)	23/126 (18.3)	0.75 (0.42, 1.32)	0.31	32/257 (12.5)	15/129 (11.6)	17/128 (13.3)	0.87 (0.46, 1.65)	0.67	0.72
6 months	43/257 (16.7)	21/128 (16.4)	22/129 (17.1)	1.18 (0.79, 1.75)	0.70	44/306 (14.4)	28/157 (17.8)	16/149 (10.7)	1.18 (0.79, 1.75)	0.11	0.15
19 months	43/256 (16.8)	26/128 (20.3)	17/128 (13.3)	1.45 (0.84, 2.49)	0.18	47/287 (16.4)	24/151 (15.9)	23/136 (16.9)	0.94 (0.57, 1.56)	0.81	0.24
12 months	43/263 (16.3)	21/136 (15.4)	22/127 (17.3)	0.87 (0.51, 1.49)	0.62	44/307 (14.3)	22/160 (13.8)	22/147 (15.0)	0.94 (0.56, 1.58)	0.81	0.85
Maternal height											
			Height <150 cm					Height ≥150 cm			
Birth	23/124 (18.5)	9/58 (15.5)	14/66 (21.2)	0.79 (0.36, 1.75)	0.57	79/606 (13.5)	48/312 (15.4)	31/294 (10.5)	1.47 (0.94, 2.28)	0.09	0.18
1 month	17/92 (18.5)	11/46 (23.9)	6/46 (13.0)	1.86 (0.74, 4.66)	0.19	51/419 (12.2)	28/206 (13.6)	23/213 (10.8)	1.19 (0.71, 1.99)	0.50	0.41
3 months	18/88 (20.5)	10/43 (23.3)	8/45 (17.8)	1.29 (0.55, 3.04)	0.56	53/434 (12.2)	21/204 (10.3)	32/209 (15.3)	0.67 (0.40, 1.11)	0.12	0.20
6 months	20/98 (20.4)	14/50 (18.0)	6/48 (12.5)	2.14 (0.90, 5.11)	0.09	67/465 (14.4)	35/235 (14.9)	32/230 (13.9)	1.01 (0.65, 1.58)	0.95	0.13
9 months	19/98 (19.4)	12/49 (24.5)	7/49 (14.3)	1.76 (0.75, 4.12)	0.19	71/445 (15.9)	38 /230 (16.5)	33/215 (15.4)	1.04 (0.68, 1.58)	0.86	0.28
12 months	15/99 (15.2)	9/52 (17.3)	6/47 (12.8)	1.40 (0.55, 3.57)	0.49	72/471 (15.3)	34/244 (13.9)	38/227 (16.7)	0.83 (0.55, 1.27)	0.39	0.32
Maternal education											
			None/Primary					Secondary /Tertiary			
Birth	74/450 (16.4)	43/232 (18.5)	31/218 (14.2)	1.32 (0.85, 2.05)	0.22	27/254 (10.6)	13/126 (10.3)	14/128 (10.9)	0.95 (0.45, 2.03)	0.90	0.47
1 month	49/325 (11.5)	27/166 (16.3)	22/159 (13.8)	1.10 (0.66, 1.84)	0.70	18/167 (10.8)	12/78 (15.4)	6/89 (6.7)	2.15 (0.85, 5.44)	0.11	0.22
3 months	51/323 (15.8)	23/164 (14.0)	28/159 (17.6)	0.79 (0.47, 1.30)	0.35	18/161 (11.2)	6/76 (7.9)	12/85 (14.1)	0.55 (0.22, 1.39)	0.21	0.51
6 months	56/350 (16.0)	31/183 (16.9)	25/167 (15.0)	1.08 (0.66, 1.77)	0.76	26/192 (13.5)	16/93 (17.2)	10/99 (10.1)	1.53 (0.74, 3.17)	0.25	0.44
9 months	56/342 (16.4)	29/180 (16.1)	27/162 (16.7)	0.95 (0.59, 1.52)	0.82	30/185 (16.2)	19/92 (20.7)	11/93 (11.8)	1.68 (0.86, 3.26)	0.13	0.17
12 months	53/354 (15.0)	28/188 (14.9)	25/166 (15.1)	1.00 (0.61, 1.64)	0.98	31/195 (15.9)	14/99 (14.1)	17/96 (17.7)	0.79 (0.42, 1.46)	0.45	0.56
Infant sex											
			Female					Male			
Birth	44/354 (12.4)	22/178 (12.4)	22/176 (12.5)	1.02 (0.57, 1.80)	0.96	58/376 (15.4)	35/192 (18.2)	23/184 (12.5)	1.48 (0.89 2.47)	0.13	0.34
1 month	30/256 (11.7)	14/125 (11.2)	16/131 (12.2)	0.93 (0.47, 1.82)	0.83	38/255 (14.9)	25/127 (19.7)	13/128 (10.2)	1.79 (0.96, 3.33)	0.07	0.16

© 2025 Mzembe G et al. *JAMA Network Open*.

eTable 3 (cont): Treatment effects on Growth and Maternal and Infant Characteristics: Subgroup analyses

	Overall Mean (SD) or n/N (%)	FCM Mean (SD) or n/N (%)	SOC Mean (SD)/ n/N (%)	Mean Difference[a] or Risk Ratio[b] (95% CI)	Two-sided P-value	Overall Mean (SD) or n/N (%)	FCM Mean (SD) or n/N (%)	SOC Mean (SD) or n/N (%)	Mean Difference[a] or Risk Ratio[b] (95% CI)	Two-sided P-value	Inter-action P-value
3 months	27/245 (11.0)	9/117 (7.7)	18/128 (14.1)	0.59 (0.28, 1.23)	0.17	44/256 (17.2)	22/130 (16.9)	22/126 (17.5)	0.92 (0.54, 1.57)	0.76	0.34
6 months	36/282 (27.8)	16/141 (11.4)	20/141 (14.2)	0.77 (0.41, 1.42)	0.39	51/281 (18.1)	33/144 (22.9)	18/137 (13.1)	1.64 (0.97, 2.77)	0.07	0.07
9 months	34/269 (12.6)	14/140 (10.0)	20/129 (15.5)	0.63 (0.34, 1.16)	0.14	56/274 (20.4.)	36/139 (25.9)	20/135 (14.8)	1.70 (1.04, 2.79)	0.04	0.01
12 months	31/281 (11.0)	13/143 (9.1)	18/138 (13.0)	0.69 (0.36, 1.34)	0.27	56/289 (19.4)	30/153 (19.6)	26/136 (19.1)	1.04 (0.65, 1.66)	0.87	0.32
**WASTING**											
Iron status at randomization											
	Iron deficient					Non-deficient					
Birth	47/259 (18.1)	23/130 (17.7)	24/129 (18.6)	1.02 (0.60, 1.71)	0.95	67/326 (20.6)	38/161 (23.6)	29/165 (17.6)	1.37 (0.88, 2.11)	0.16	0.39
1 month	26/198 (13.1)	17/100 (17.0)	9/98 (9.2)	1.86 (0.88, 3.95)	0.11	32/279 (11.5)	14/131 (10.7)	18/148 (12.2)	0.88 (0.47, 1.64)	0.68	0.13
3 months	20/194 (10.3)	12/96 (12.5)	8/98 (8.2)	1.61 (0.70, 3.74)	0.26	25/288 (8.7)	10/140 (7.1)	15/148 (10.1)	0.73 (0.35, 1.52)	0.40	0.16
6 months	25/225 (11.1)	18/112 (16.1)	7/113 (6.2)	2.55 (1.11, 5.83)	0.03	29/320 (9.1)	14/163 (8.6)	15/157 (9.6)	0.89 (0.44, 1.80)	0.75	0.06
9 months	27/222 (12.2)	18/115 (15.7)	9/107 (8.4)	1.88 (0.91, 3.87)	0.09	27/306 (8.8)	15/154 (9.7)	12/152 (7.9)	1.27 (0.91, 3.87)	0.52	0.45
12 months	33/236 (14.0)	13/123 (10.6)	20/113 (17.7)	0.59 (0.32, 1.08)	0.09	38/319 (11.9)	19/163 (11.7)	19/156 (12.2)	1.01 (0.58, 1.78)	0.96	0.20
Inflammation at randomization											
	Yes					No					
Birth	64/301 (21.3)	34/145 (23.5)	30/156 (19.2)	1.31 (0.83, 2.06)	0.25	38/347 (11.0)	20/175 (11.4)	18/172 (10.5)	1.12 (0.69, 1.84)	0.64	0.66
1 month	31/245 (12.7)	16/122 (13.1)	15/125 (12.0)	1.11 (0.58, 2.09)	0.76	27/230 (11.7)	15/109 (13.8)	12/121 (9.9)	1.36 (0.68, 2.73)	0.39	0.67
3 months	21/245 (8.6)	8/121 (6.6)	13/124 (10.5)	0.69 (0.30, 1.61)	0.39	24/237 (10.1)	14/115 (12.2)	10/122 (8.2)	1.46 (0.69, 3.09)	0.32	0.19
6 months	34/279 (12.2)	20/142 (14.1)	14/137 (10.2)	1.38 (0.73, 2.63)	0.32	20/266 (7.5)	12/133 (9.0)	8/133 (6.0)	1.46 (0.61, 3.45)	0.39	0.93
9 months	21/267 (7.9)	14/137 (10.2)	7/130 (5.4)	2.06 (0.88, 4.82)	0.46	33/261 (12.6)	19/132 (14.4)	14/129 (10.9)	1.26 (0.68, 2.36)	0.10	0.37
12 months	30/275 (10.9)	12/143 (8.4)	18/132 (13.6)	0.66 (0.34, 1.29)	0.22	41/280 (14.6)	20/143 (14.0)	21/137 (15.3)	0.89 (0.53, 1.50)	0.66	0.49
Placental malaria											
	Positive					Negative					
Birth	38/179 (21.2)	17/88 (19.3)	21/91 (23.1)	1.11 (0.62, 1.98)	0.72	77/418 (18.4)	36/208 (17.3)	41/210 (19.5)	1.23 (0.82, 1.85)	0.32	0.78
1 month	14/150 (9.3)	10/79 (12.7)	4/71 (5.6)	0.41 (0.14, 1.19)	0.10	45/342 (13.2)	17/172 (9.9)	28/170 (16.5)	1.72 (1.00, 2.99)	0.053	0.02
3 months	14/158 (8.9)	9/81 (11.1)	5/77 (6.5)	0.56 (0.20, 1.56)	0.39	31/33 (9.1)	14/170 (8.2)	17/169 (10.1)	1.32 (0.69, 2.54)	0.41	0.17
6 months	18/183 (9.8)	9/90 (10.0)	9/93 (9.7)	0.92 (0.39, 2.20)	0.86	37/378 (9.8)	13/186 (7.0)	24/192 (12.5)	1.80 (0.94, 3.45)	0.08	0.23
9 months	16/180 (8.9)	6/87 (6.9)	10/93 (10.8)	1.41 (0.55, 3.61)	0.47	38/363 (10.5)	15/177 (8.5)	23/186 (12.4)	1.56 (0.86, 2.83)	0.15	0.87
12 months	21/184 (11.4)	8/86 (9.3)	13/98 (13.3)	1.30 (0.58, 2.95)	0.52	50/386 (12.9)	31/188 (16.4)	19/198 (09.6)	0.62 (0.38, 1.02)	0.060	0.13
Maternal age											
	Age <20					Age ≥20 years					
Birth	58/260 (22.3)	31/130 (23.9)	27/130 (20.8)	1.27 (0.80, 2.01)	0.32	57/337 (16.9)	31/171 (18.1)	26/166 (15.7)	1.18 (0.74, 1.89)	0.49	0.83
1 month	24/233 (10.3)	8/108 (7.4)	16/125 (12.8)	0.62 (0.28, 1.34)	0.22	35/259 (13.5)	24/133 (18.1)	11/126 (8.7)	2.00 (1.04, 3.86)	0.04	0.02
3 months	15/242 (6.2)	7/118 (5.9)	8/124 (6.5)	0.99 (0.37, 2.61)	0.98	30/255 (11.8)	15/128 (11.7)	15/127 (11.8)	1.02 (0.53, 1.97)	0.95	0.95
6 months	24/256 (9.4)	13/128 (10.2)	11/128 (8.6)	1.20 (0.56, 2.58)	0.64	31/305 (10.2)	20/157 (12.7)	11/148 (7.4)	1.68 (0.83, 3.38)	0.15	0.53
9 months	23/256 (9.0)	15/128 (11.7)	8/128 (6.3)	1.94 (0.86, 4.36)	0.11	31/287 (10.8)	18/151 (11.9)	13/136 (9.6)	1.28 (0.67, 2.43)	0.46	0.43
12 months	29/263 (11.0)	14/136 (10.3)	15/127 (11.8)	0.89 (0.46, 1.75)	0.74	42/307 (13.7)	18/160 (11.3)	24/147 (16.3)	0.71 (0.42, 1.19)	0.19	0.59
Maternal height											
	Height <150 cm					Height ≥150 cm					
Birth	22/94 (23.4)	11/48 (22.9)	11/46 (23.9)	1.20 (0.51, 2.34)	0.81	93/503 (18.5)	51/253 (20.2)	42/250 (16.8)	1.24 (0.85, 1.79)	0.26	0.78
1 month	12/87 (13.8)	9/42 (21.4)	3/45 (6.7)	3.38 (1.03, 11.15)	0.05	27/405 (6.7)	23/199 (11.6)	24/206 (11.7)	0.98 (0.58, 1.66)	0.95	0.06
3 months	7/87 (8.0)	4/43 (9.3)	3/44 (6.8)	1.59 (0.37, 6.80)	0.53	38/410 (9.3)	18/203 (8.9)	20/207 (9.7)	0.94 (0.52, 1.70)	0.84	0.51
6 months	10/98 (10.2)	8/50 (16.0)	2/48 (4.2)	4.21 (0.93, 19.02)	0.06	45/463 (9.7)	25/235 (10.6)	20/228 (8.8)	1.18 (0.67, 2.07)	0.56	0.12
9 months	6/98 (6.1)	4/49 (8.2)	2/49 (4.1)	2.28 (0.44, 11.85)	0.33	48/445 (10.8)	29/230 (12.6)	19/215 (8.8)	1.43 (0.84, 2.42)	0.19	0.60
12 months	12/99 (12.1)	5/52 (9.6)	7/47 (14.9)	0.77 (0.23, 2.22)	0.63	59/471 (12.5)	27/244 (11.1)	32/227 (14.1)	0.78 (0.50, 1.22)	0.28	0.99
Maternal education											

© 2025 Mzembe G et al. *JAMA Network Open*.

eTable 3 (cont): Treatment effects on Growth and Maternal and Infant Characteristics: Subgroup analyses

	Overall Mean (SD) or n/N (%)	FCM Mean (SD) or n/N (%)	SOC Mean (SD)/ n/N (%)	Mean Difference[a] or Risk Ratio[b] (95% CI)	Two-sided P-value	Overall Mean (SD) or n/N (%)	FCM Mean (SD) or n/N (%)	SOC Mean (SD) or n/N (%)	Mean Difference[a] or Risk Ratio[b] (95% CI)	Two-sided P-value	Inter-action P-value
	None/Primary					Secondary/Tertiary					
Birth	79/353 (22.4)	43/185 (23.2)	36/168 (21.4)	1.15 (0.79, 1.69)	0.46	34/223 (15.2)	18/107 (16.8)	16/116 (13.8)	1.22 (0.65, 2.29)	0.54	0.88
1 month	36/311 (11.6)	19/158 (12.0)	17/153 (11.1)	1.12 (0.61, 2.04)	0.72	20/162 (12.3)	12/75 (16.0)	8/87 (9.2)	1.56 (0.69, 3.55)	0.29	0.52
3 months	28/321 (8.7)	12/164 (7.3)	16/157 (10.2)	0.76 (0.38, 1.54)	0.45	15/159 (9.4)	9/75 (12.0)	7/84 (8.3)	1.46 (0.58, 3.66)	0.42	0.27
6 months	30/349 (8.6)	18/183 (9.8)	12/166 (7.2)	1.41 (0.69, 2.87)	0.35	23/191 (12.0)	14/93 (15.1)	9/98 (9.2)	1.50 (0.68, 3.30)	0.32	0.91
9 months	30/342 (8.8)	17/180 (9.4)	13/162 (8.0)	1.24 (0.63, 2.46)	0.54	24/185 (13.0)	16/92 (17.4)	8/93 (8.6)	1.98 (0.94, 4.17)	0.07	0.37
12 months	40/354 (11.3)	18/188 (9.6)	22/166 (13.3)	0.79 (0.45, 1.39)	0.41	27/195 (13.8)	12/99 (12.1)	15/96 (15.6)	0.72 (0.38, 1.37)	0.32	0.83
Maternal HIV status											
	Positive					Negative					
Birth	18/123 (14.6)	12/61 (19.7)	6/62 (9.7)	1.08 (0.52, 2.21)	0.84	92/486 (18.9)	50/246 (20.3)	42/240 (17.5)	1.24 (0.85, 1.80)	0.27	0.74
1 month	8/80 (10.0)	6 /44(13.6)	2/36 (5.6)	1.57 (0.56, 4.42)	0.39	46/410 (11.2)	23/196 (11.7)	23/214 (10.8)	1.13 (0.66, 1.92)	0.66	0.58
3 months	10/81 (12.3)	8/42 (19.1)	2/39 (5.1)	1.82 (0.47, 7.06)	0.39	36/412 (8.74)	16/203 (7.9)	20/209 (9.6)	0.87 (0.47, 1.61)	0.66	0.34
6 months	22/98 (22.4)	14/54 (25.9)	8/44 (18.2)	1.70 (0.57, 5.11)	0.34	41/459 (8.9)	23/230 (10.0)	18/229 (7.9)	1.33 (0.73, 2.39)	0.35	0.69
9 months	26/97 (26.8)	16/53 (30.2)	10/44 (22.7)	1.25 (0.46, 3.39)	0.66	41/442 (9.3)	25/225 (11.1)	16/217 (7.4)	1.58 (0.88, 2.84)	0.12	0.69
12 months	20/100 (20.1)	11/52 (21.2)	9/48 (18.8)	1.34 (0.57, 3.15)	0.51	56/466 (12.0)	23/243 (9.5)	33/223 (14.8)	0.66 (0.41, 1.07)	0.10	0.16
Infant sex											
		Female				Male					
Birth	56/290 (19.3)	30/143 (21.0)	26/147 (17.7)	1.27 (0.80, 2.04)	0.31	59/307 (19.2)	32/158 (20.3)	27/149 (18.1)	1.15 (0.72, 1.83)	0.57	0.75
1 month	29/246 (22.8)	16/119 (13.5)	13/127 (10.2)	1.43 (0.73, 2.81)	0.30	30/246 (12.2)	16/122 (13.1)	14/124 (11.3)	1.07 (0.56, 2.06)	0.83	0.55
3 months	23/243 (9.5)	9/116 (7.8)	14/127 (11.0)	0.78 (0.36, 1.70)	0.53	22/154 (14.3)	13/130 (10.0)	9/124 (7.3)	1.37 (0.62, 3.04)	0.44	0.32
6 months	28/281 (10.0)	14/141 (9.9)	14/140 (10.0)	1.05 (0.52, 2.13)	0.89	27/280 (9.6)	19/144 (13.2)	8/136 (5.9)	2.10 (0.94, 4.64)	0.07	0.20
9 months	19/269 (7.1)	10/140 (7.1)	9/129 (7.0)	1.05 (0.46, 2.43)	0.90	35/274 (12.8)	23/139 (16.6)	12/135 (8.9)	1.88 (0.99, 3.55)	0.05	0.28
12 months	32/281 (11.4)	13/143 (9.1)	19/138 (13.8)	0.67 (0.35, 1.25)	0.21	39/289 (13.5)	19/153 (12.4)	20/136 (14.7)	0.88 (0.51, 1.52)	0.64	0.52

Abbreviations: FCM, Ferric carboxymaltose; SOC, Standard-of-care; SD, standard deviation; CI, confidence interval.

Includes all liveborn infants from the REVAMP trial whose mothers consented to participate in the extended follow up and with at least one non-missing outcome value. Of the mothers who consented to their infants participating in the follow-up up to 12 months of age, 17/755 (2.3%) did not have data available at any one point in time, thus, a total of 738 were included.

[a] Mean difference: An absolute mean difference of IV FCM versus SOC, at birth, 1, 3, 6, 9, and 12 months of age is shown following analyses using a longitudinal data analysis model (including all study visits from birth to 12 months) with a random intercept for participants and an unstructured variance-covariance among the repeated measurements. Subgroup (fixed effect) and subgroup-by-treatment-by-visit interaction (and subgroup-by-treatment and subgroup-by-visit interaction) have been added to the model to evaluate how the treatment effect differs between subgroup categories.

[b] Risk ratio: A risk ratio of IV FCM versus SOC for stunting, wasting and underweight at birth, 1, 3, 6, 9, and 12 months of age is shown following analyses using a Poisson model with random intercept for the infants and robust standard errors. Subgroup (fixed effect) and subgroup-by-treatment-by-visit interaction (and subgroup-by-treatment and subgroup-by-visit interaction) have been added to the model to evaluate how the treatment effect differs between subgroup categories.

© 2025 Mzembe G et al. *JAMA Network Open*.
