# Supplementary material for: Prenatal Intravenous Iron and Child Growth: A Secondary Analysis of a Randomized Clinical Trial
Source: JAMA Netw Open. 2025 Oct 28;8(10):e2538392. doi: 10.1001/jamanetworkopen.2025.38392 (PMC12569709; doi:10.1001/jamanetworkopen.2025.38392)
Supplement: Supplement 3. — Data Sharing Statement [file jamanetwopen-e2538392-s003.pdf]

Data Sharing Statement

Mzembe. Prenatal Intravenous Iron and Child Growth. *JAMA Netw Open*. Published October 28, 2025. doi:10.1001/jamanetworkopen.2025.38392

Data
Additional Information: The Australian New Zealand Clinical Trials Registry ACTRN12618001268235.
Data available: Yes
Data types: Deidentified participant data, Data dictionary
How to access data: Underlying deidentified individual participant data encompassing the reported trial results and a data dictionary are accessible at figshare (https://figshare.unimelb.edu.au/articles/dataset/Prenatal_Intravenous_Iron_and_Child_Growth_A_secondary_analysis_of_a_Randomized_Clinical_Trial/3[illegible]
Files are embargoed until publication. Data are available under the terms of Creative Commons Attribution 4.0 International License (CC-BY-4.0).
When available: With publication

Supporting Documents
Document types: Informed consent form
How to access documents: These are available by the author (Glory Mzembe: gmzembe@cartafrica.org) upon reasonable request.
When available: With publication

Additional Information
Who can access the data: The de-identified data are available under the terms of Creative Commons Attribution 4.0 International License (CC-BY-4.0).
Types of analyses: For any purpose.
Mechanisms of data availability: To maintain the privacy and confidentiality of the research participants, access is provided to de-identified data only. Certain variables have not been included due to their risk of re-identification, as outlined in the data dictionaries. These variables are available by the author (Rebecca Harding: harding.r@wehi.edu.au) upon reasonable request.
